# Lipin1 depletion coordinates neuronal signaling pathways to promote motor and sensory axon regeneration after spinal cord injury

**DOI:** 10.1073/pnas.2404395121

**Published:** 2024-09-18

**Authors:** Weitao Chen, Junqiang Wu, Chao Yang, Suying Li, Zhewei Liu, Yongyan An, Xuejie Wang, Jiaming Cao, Jiahui Xu, Yangyang Duan, Xue Yuan, Xin Zhang, Yiren Zhou, Jacque Pak Kan Ip, Amy K. Y. Fu, Nancy Y. Ip, Zhongping Yao, Kai Liu

**Affiliations:** ^a^Biomedical Research Institute, Shenzhen Peking University–The Hong Kong University of Science and Technology Medical Center, Shenzhen 518036, China; ^b^Division of Life Science, State Key Laboratory of Molecular Neuroscience, The Hong Kong University of Science and Technology, Hong Kong, China; ^c^Hong Kong Center for Neurodegenerative Diseases, Hong Kong, China; ^d^Guangdong Provincial Key Laboratory of Brain Science, Disease and Drug Development, Hong Kong University of Science and Technology Shenzhen Research Institute, Shenzhen-Hong Kong Institute of Brain Science, Shenzhen, Guangdong 518057, China; ^e^State Key Laboratory of Chemical Biology and Drug Discovery, Research Institute for Future Food, Research Centre for Chinese Medicine Innovation, The Hong Kong Polytechnic University, Hung Hom, Kowloon, Hong Kong Special Administrative Region, China; ^f^Department of Applied Biology and Chemical Technology, The Hong Kong Polytechnic University, Hung Hom, Kowloon, Hong Kong Special Administrative Region, China; ^g^State Key Laboratory of Chinese Medicine and Molecular Pharmacology (Incubation), Hong Kong Polytechnic University Shenzhen Research Institute, Shenzhen 518057, China; ^h^Shenzhen Key Laboratory of Food Biological Safety Control, Hong Kong Polytechnic University Shenzhen Research Institute, Shenzhen 518057, China; ^i^School of Biomedical Sciences, The Chinese University of Hong Kong, Hong Kong Special Administrative Region, China; ^j^Department of Chemical and Biological Engineering, The Hong Kong University of Science and Technology, Hong Kong, China

**Keywords:** axon regeneration, spinal cord injury, lipid metabolism, lipid signaling, lipin1

## Abstract

Adult central nervous system (CNS) neurons down-regulate growth programs after injury, leading to persistent regeneration failure. Coordinated lipids metabolism is required to synthesize membrane components during axon regeneration. However, lipids also function as cell signaling molecules. Whether lipid signaling contributes to axon regeneration remains unclear. In this study, we showed that lipin1 orchestrates mechanistic target of rapamycin (mTOR) and STAT3 signaling pathways to determine axon regeneration. We established an mTOR-lipin1-phosphatidic acid/lysophosphatidic acid-mTOR loop that acts as a positive feedback inhibitory signaling, contributing to the persistent suppression of CNS axon regeneration following injury. In addition, lipin1 knockdown (KD) enhances corticospinal tract (CST) sprouting after unilateral pyramidotomy and promotes CST regeneration following complete spinal cord injury (SCI). Furthermore, lipin1 KD enhances sensory axon regeneration after SCI. Overall, our research reveals that lipin1 functions as a central regulator to coordinate mTOR and STAT3 signaling pathways in the CNS neurons and highlights the potential of lipin1 as a promising therapeutic target for promoting the regeneration of motor and sensory axons after SCI.

## Significance

Traumatic injuries to the central nervous system (CNS) in mammals often lead to permanent functional impairments because the CNS has limited regeneration capacity. Despite recent progress, achieving significant regrowth of nerve fibers after a complete spinal cord injury (SCI) remains a major challenge. Our previous finding demonstrates that inhibiting lipin1, a phosphatidic acid phosphatase, rewires lipid metabolism to promote axon regeneration. Here, we revealed that lipin1 modulates axon regeneration capacity by influencing cell signaling pathways critical for axon regeneration. Deleting lipin1 facilitates robust regrowth of multiple CNS axons following injury. This finding suggests a potential strategy for repairing the damaged neural network after spinal cord injury, offering hope for future clinical applications.

Limited axon regeneration in the central nervous system (CNS) of adult mammals leads to permanent functional deficits after injury. Therefore, understanding the underlying mechanisms that restore axon regeneration and reestablish neural networks is crucial. During axon regeneration, a substantial amount of lipids is required for membrane assembly (1–3). Upon injury, CNS neurons tend to synthesize triglycerides for energy storage. Inhibiting lipin1, a critical phosphatidic acid phosphatase (PAP) enzyme, redirects lipid metabolism to synthesize more membrane-component phospholipids (PLs), facilitating axon regeneration (4). Nevertheless, lipids also play critical roles in various cell signaling processes (5–8). One such signaling lipid is phosphatidic acid (PA), the substrate for lipin1. PA is a central precursor for phospholipid synthesis (9, 10). Furthermore, PA binds to a conserved sequence within the α4 helix of the FK506 binding protein 12/rapamycin-binding domain of mTOR, modulating substrate binding and the catalytic activity of mTOR complex 1 (mTORC1) (11, 12). Another well-documented signaling lipid is lysophosphatidic acid (LPA), which engages in reciprocal conversion with PA in the glycerol phosphate pathway (8). LPA signals through the specific G protein-coupled receptors (GPCRs) and intracellular receptor peroxisome proliferator-activated receptor-gamma (PPARγ), regulating cell proliferation, migration, and cytoskeletal reorganization (13, 14). Therefore, it is crucial to elucidate the contribution of lipid signaling to axon regeneration.

The distinct reactions of neurons in the CNS and peripheral nervous system (PNS) to traumatic injury contribute to the differences observed in axon regeneration (15–24). Evidence has shown that the growth state is constantly suppressed in injured CNS neurons. For instance, mTOR activity in alpha retinal ganglion cells (RGCs) and corticospinal motor neurons declines shortly after axon injury (25, 26). mTOR activation by stimulating neuronal activity with the designer receptor exclusively activated by a designer drug (DREADD-Gq) was sustained for a shorter time in injured RGCs than in intact RGCs, suggesting the presence of an injury-induced mechanism that suppresses mTOR (27). Notably, reactivation of mTOR through the deletion of phosphatase and tensin homolog (PTEN) enhances corticospinal tract regeneration, even 1 y after spinal cord injury (SCI) (28). Several studies have revealed a conserved role of the PTEN/mTOR pathway in regulating axon regeneration across vertebrate and invertebrate animals (29–33). Despite growing evidence highlighting the importance of mTOR, the downregulation of mTOR in injured CNS neurons is poorly understood.

The JAK/STAT3 pathway is a crucial regulator of axon regeneration in response to traumatic injury. Following peripheral injury, the signal transducer and activator of transcription 3 (STAT3) is activated and required for axon regeneration (34, 35). In contrast, this process is notably absent in the CNS. Stimulating the JAK/STAT3 pathway has been shown to increase the regenerative capacity of CNS axons (36–38). Interestingly, the coactivation of mTOR and STAT3 signaling pathways exhibits a synergistic effect on axon regeneration (39, 40). However, it is yet to be elucidated whether there is a communal regulator of mTOR and STAT3 signaling pathways in CNS neurons.

In recent decades, our understanding of CNS axon regeneration has significantly advanced. Various genes within the neurons, including PTEN (25, 28), suppressor of cytokine signaling 3 (40), Krüppel-like factors (41), SOX11 (42), LKB1 (43), Lin28 (44), and B-Raf (45), have been demonstrated to control the regrowth of spinal axons following SCI. Nevertheless, promoting axon regeneration of both the descending motor and the ascending sensory axons after SCI has continued to be a difficult task. Hence, it is crucial to investigate new target genes that facilitate the regeneration of axons in various types of neurons after SCI. In this study, we provide evidence for the pivotal role of lipin1 in orchestrating the mTOR and STAT3 signaling pathways, thereby influencing CNS axon regeneration. Inhibiting lipin1 expression dramatically enhanced the regenerative capacity of the corticospinal tract (CST) and sensory axons following SCI. These findings establish lipin1 as an important modulator of axon regeneration across diverse cell types.

## Results

### Lipin1 Orchestrates mTOR and STAT3 Signaling to Render Axon Regeneration.

**Fig. 1.**
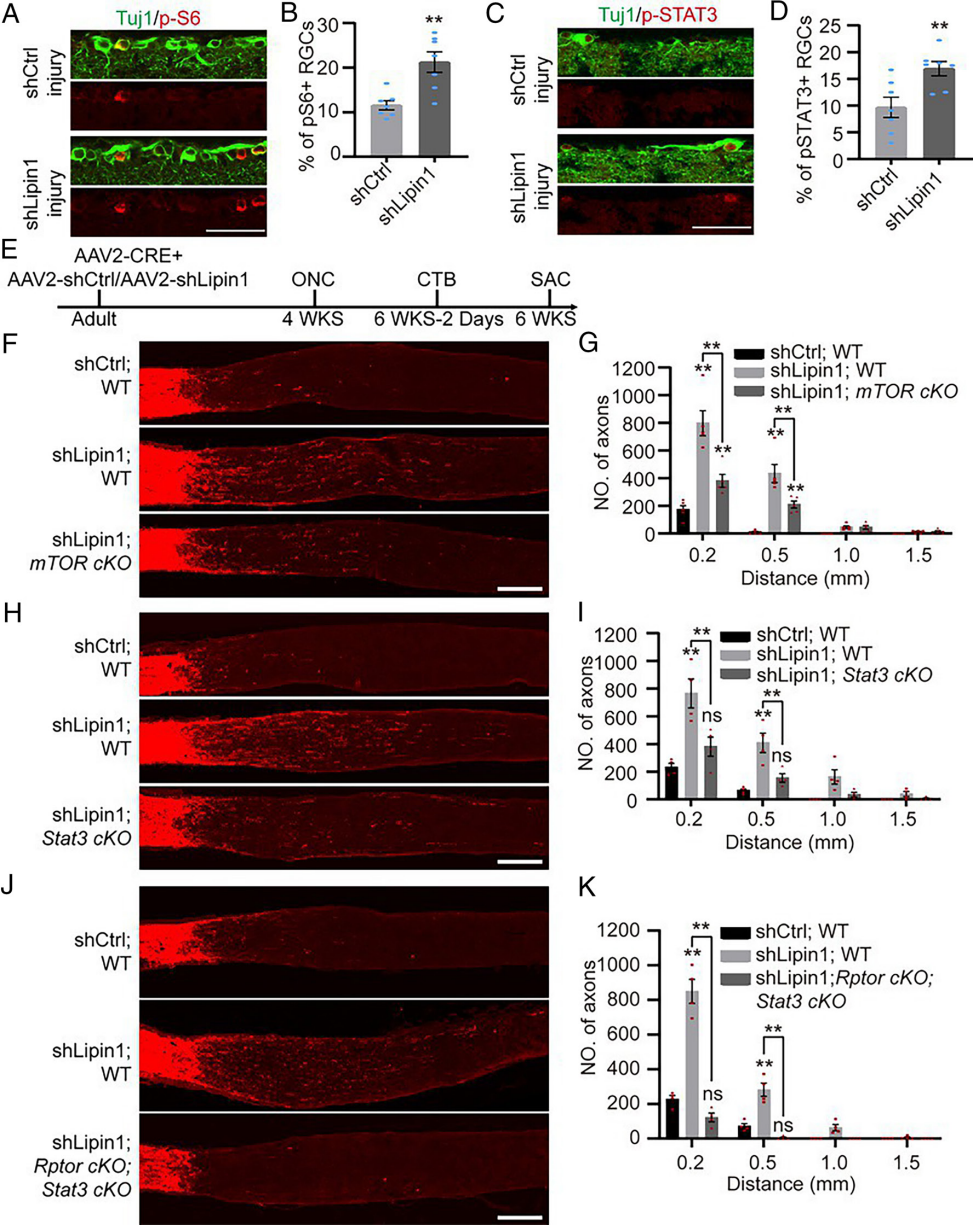
Lipin1 KD up-regulates mTORC1 and STAT3 signaling to boost axon regeneration. (*A*–*D*) Phospho-S6 (*A* and *B*) and p-STAT3 (*C* and *D*) activation in Ctrl and Lipin1 KD RGCs. (Scale bar: 50 μm.) **P ≤ 0.01, Student’s *t* test. n = 7 mice. (*E*) Experiment schematic. (*F* and *G*) Axon regeneration with indicated treatments (*F*) and quantification (*G*). (Scale bar: 200 μm.) **P ≤ 0.01, ANOVA followed by Šídák’s test, n = 5 mice. (*H* and *I*) Axon regeneration with indicated treatments (*H*) and quantification (*I*). (Scale bar: 200 μm.) **P ≤ 0.01, ns, not significant, ANOVA followed by Šídák’s test, n = 4 mice. (*J* and *K*) Axon regeneration with indicated treatments (*J*) and quantification (*K*). (Scale bar: 200 μm.) **P ≤ 0.01, ns, not significant, ANOVA followed by Šídák’s test, n = 4 mice. See also *SI Appendix*, Fig. S1.

Lipin1 suppression directs glycerolipid metabolism to PLs synthesis for membrane extension and promotes axon regeneration (4). Nevertheless, lipids also participate in cell signaling. It remains unclear whether cell signaling plays a role in lipin1 inhibition-induced axon regeneration. To address this question, we examined several well-established signaling pathways critical for axon regeneration, including mTOR, STAT3, extracellular signal-regulated kinase (ERK), and Akt. We designed short-hairpin RNA (shRNA) that targets lipin1 and used serotype 2 adeno-associated virus (AAV2) to deliver scrambled shRNA (shCtrl) or shLipin1 into the eyes of WT mice. We performed an optic nerve injury 4 wk later and collected samples 1 d later to examine the RGCs’ response to the injury. The retinas received whole mount staining of Tuj1 to indicate the RGCs, and the GFP expression indicated AAV2 infection. Our AAV2 infected 89% of the RGCs (*SI Appendix*, Fig. S1 *A* and *B*). The sectioned retinas were immunostained with cell signaling markers. We observed more RGCs expressing p-S6 and p-STAT3 in the shLipin1 group compared with the shCtrl group (Fig. 1 *A*–*D*). However, p-AKT473 and p-ERK levels did not change following lipin1 KD (*SI Appendix*, Fig. S1 *C*–*F*). These data suggest that lipin1 KD boosts mTOR and STAT3 signaling.

We next asked whether the mTOR and STAT3 activity was involved in lipin1 KD-induced axon regeneration. We first investigated the function of mTOR. AAV2-Cre, along with AAV2-shCtrl or AAV2-shLipin1, was intravitreally infused into the eyes of WT and *mTOR*^f/f^ mice, followed by an optic nerve crush (ONC) 4 wk later. CTB-FITC was delivered into the eyes to trace the optic nerve 2 d before killing (Kil) the animals (Fig. 1*E*). The sagittal section of the optic nerve was immunostained with FITC to visualize the axons. Consistent with the previous study (4), very few axons were regenerated in the control group, and lipin1 KD led to extensive axon regeneration (Fig. 1 *F* and *G*). In contrast, *mTOR* cKO substantially reduced the axon regeneration (Fig. 1 *F* and *G*). We assessed RGC survival by Tuj1 staining of the retinas and found no differences among the three groups (*SI Appendix*, Fig. S1*G*). *mTOR* cKO abolished the p-S6 expression (*SI Appendix*, Fig. S1*H*), indicating successful deletion of *mTOR*. These results suggest that lipin1 suppresses the regenerative capacity of the optic nerve partially by inhibiting mTOR activity.

mTOR primarily regulates the ability of axon regeneration through mTORC1 (46, 47). Next, we examined whether mTORC1 contributes to the lipin1 KD-induced axon regeneration. mTOR Regulatory–associated protein of mammalian target of rapamycin (RPTOR) is the scaffold protein of mTORC1, which is known to play a crucial role in axon regeneration (46, 48). We utilized WT and *Rptor*^f/f^ mice and followed the experiment procedure indicated in Fig. 1*E*. Lipin1 KD facilitated much weaker axon regeneration in the *Rptor* cKO mice than the WT mice (*SI Appendix*, Fig. S1 *I* and *J*). The RGC survival was not altered by *Rptor* cKO (*SI Appendix*, Fig. S1*K*). The elimination of p-S6 indicated a successful *Rptor* cKO (*SI Appendix*, Fig. S1*L*). Therefore, our data suggest that lipin1 KD required mTORC1 activity to promote axon regeneration.

Since STAT3 is also activated following lipin1 KD, we next explored the role of STAT3 signaling in lipin1 KD-mediated axon regeneration. We utilized WT and *Stat3*^f/f^ mice and followed the experiment procedure indicated in Fig. 1*E*. The animals with lipin1 KD and *Stat3* cKO displayed weaker axon regeneration than those with lipin1 KD only (Fig. 1 *H* and *I*). The RGC survival in the three groups was similar (*SI Appendix*, Fig. S1*M*). The STAT3 phosphorylation diminished in the RGCs with *Stat3* cKO (*SI Appendix*, Fig. S1*N*), indicating a successful deletion of STAT3. These findings collectively suggest that STAT3 activation is required for axon regeneration induced by lipin1 suppression.

As axon regeneration following lipin1 KD is partially suppressed by either *mTOR* or *Stat3* cKO, we aimed to investigate the combined effects of mTOR and STAT3. To simultaneously disrupt mTORC1 and STAT3 signaling, we crossed the *Rptor*^f/f^ and *Stat*3^f/f^ mice and administered AAV2-Cre into their eyes. Consistently, lipin1 KD induced significant axon regeneration, whereas *Rptor* and *Stat3* double cKO blocked the axon regeneration (Fig. 1 *J* and *K*). The double cKO group showed comparable RGC survival with the other two groups (*SI Appendix*, Fig. S1*O*). The absence of both p-S6 and p-STAT3 in the RGCs confirmed the successful knockout of both genes (*SI Appendix*, Fig. S1 *P* and *Q*). Overall, these findings demonstrate that lipin1 coordinates mTOR and STAT3 signaling pathways to promote axon regeneration.

### PA and LPA Mediate the Activation of mTOR and STAT3 Following Lipin1 KD.

**Fig. 2.**
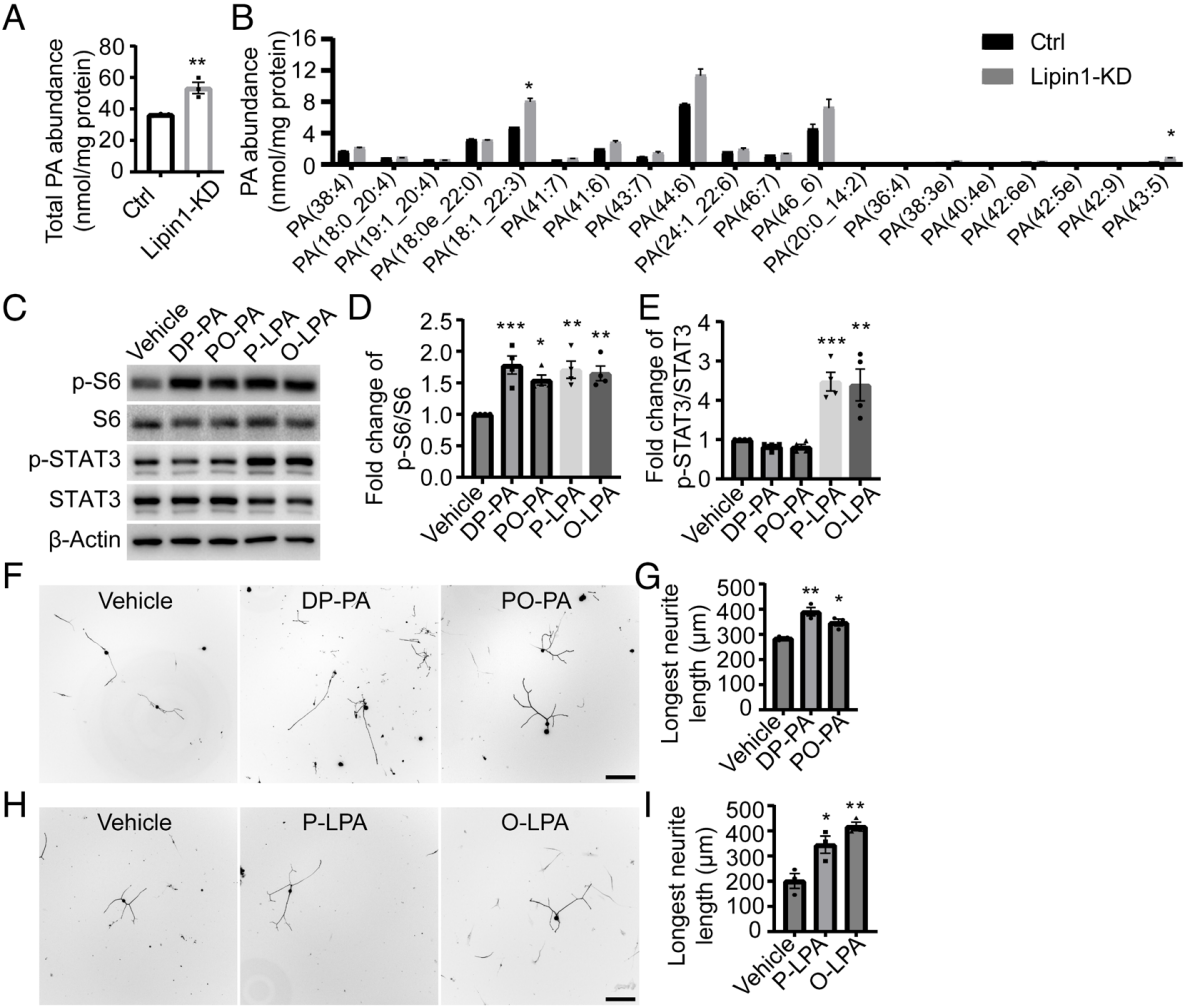
The PA and LPA activate cell signaling pathways after lipin1 KD. (*A*) Total PA level in the cortical neurons infected with AAV9-GFP or AAV9-shLipin1. **P ≤ 0.01, Student’s *t* test. n = 3. (*B*) The abundance of individual PA species in the cortical neurons infected with AAV9-GFP or AAV9-shLipin1. *P ≤ 0.05, *t* test, n = 3. (*C*–*E*) WB analysis of p-S6 and p-mTOR in the cultured DRG neurons with the vehicle, DP-PA, or PO-PA treatment. *P ≤ 0.05, **P ≤ 0.01, ***P ≤ 0.001, ANOVA followed by Dunnett’s test. n = 4. (*F* and *G*) Neurites regrowth of DRG neurons with the vehicle, DP-PA, or PO-PA treatment (*F*) and quantification of longest neurite length (*G*). (Scale bar: 200 μm.) *P ≤ 0.05, **P ≤ 0.01, ANOVA followed by Dunnett’s test, n = 3. (*H* and *I*) Neurites regrowth of DRG neurons with the vehicle, P-LPA, or O-LPA treatment (*H*) and quantification of longest neurite length (*I*). (Scale bar: 200 μm.) *P ≤ 0.05, **P ≤ 0.01, ANOVA followed by Dunnett’s test, n = 3. See also *SI Appendix*, Fig. S2.

We next explored the mechanism underlying the fact that lipin1 functions as an inhibitor of mTOR and STAT3 in injured RGCs. One potential mediator is PA, which has been reported to accumulate in lipin1-deficient mice and enhance substrate binding and the catalytic activity of mTORC1 by directly binding to mTOR (11, 49). Therefore, we examined whether PA concentrations changed in neurons following lipin1 KD. We cultured E18 cortical neurons and infected them with either AAV9-shCtrl or AAV9-shLipin1. Lipids were extracted and analyzed using an ultraperformance liquid chromatography–mass spectrometry (UPLC-MS) system. We identified 20 subspecies of PA, the total abundance of which increased by 47% after lipin1 KD (Fig. 2*A*). Notably, the levels of PA (18:1_22:3) and PA (43:5) exhibited a significant increase (Fig. 2*B*). In addition, LPA level also increased after lipin1 KD and contributes to various cell signaling (4, 13, 14). Thus, we next investigated whether exposure to PA and LPA was sufficient to affect mTOR and STAT3 activity. We employed two commercially available PA species, 1,2-dipalmitoyl-sn-glycero-3-phosphate (DP-PA) and 1-palmitoyl-2-pleoyl-sn-glycero-3-phosphatidic acid (PO-PA), and two commercially available LPA species, 1-palmitoyl-lyso-phosphatidic acid Na salt (P-LPA) and 1-oleoyl-lyso-phosphatidic acid Na salt (O-LPA). We encapsulated them into lipid vesicles using a previously described protocol to enhance intracellular delivery (50). Dorsal root ganglion (DRG) neurons isolated from adult animals were cultured for 4 d, and the PA- and LPA-containing lipid vesicles were added to the culture medium. After 30 min, the DRG neurons were collected for Western Blots (WB) analysis. Both PA treatments increased p-S6 but not p-STAT3 expression (Fig. 2 *C*–*E*). Interestingly, both LPAs elevate expression levels of p-S6 and p-STAT3 (Fig. 2 *C*–*E*). These observations indicate PA and LPA contribute to the activation of mTOR and STAT3 after lipin1 KD.

Then, we investigated whether PA and LPA were sufficient to promote axon growth with the widely used DRG culture system. We cultured adult DRG neurons and applied lipid vesicles containing either vehicle, PAs, or LPAs in the culture medium daily, from day 1 to day 4. On day 5, the DRG neurons were replated in a new 24-well plate and allowed to grow for 18 to 20 h. Immunostaining for Tuj1 was performed to visualize the neurites of the DRG neurons. Administration of DP-PA and PO-PA resulted in a 37% and 22% increase in DRG neurite outgrowth, respectively (Fig. 2 *F* and *G*). Moreover, P-LPA and O-LPA delivery enhanced the neurite outgrowth by 71% and 108%, respectively (Fig. 2 *H* and *I*). These data suggest that neuronal delivery of PA and LPA are capable of promoting neurite outgrowth.

Despite using lipid vesicles to enhance intracellular delivery of PA and LPA, we cannot rule out the possibility that PA and LPA may impact axon growth via extracellular receptors. To address this issue, we directly added PA and LPA to the culture media and assessed their impact on axon growth. Notably, direct PA treatment did not promote axon elongation (*SI Appendix*, Fig. S2*A*). Conversely, exposure to unencapsulated LPA resulted in longer axons compared to the control group, although these neurites were much shorter than those found in the lipid vesicle-encapsulated LPA group (*SI Appendix*, Fig. S2*B*). To determine whether LPA receptors were involved in LPA-induced axon growth, we used three well-studied LPA receptor antagonists to inhibit LPA_1_ and LPA_3_ receptors (51–53). The antagonists did not affect neurite outgrowth, whether in the presence or absence of LPA treatment (*SI Appendix*, Fig. S2*C*), indicating that these LPA receptors are not necessary for LPA-induced axon growth. LPA_1-5_ receptors’ signaling can also be activated by a synthetic compound AzoLPA, which shifts between isoforms under different light conditions. The *cis*-AzoLPA form, induced by 365 nm light, has a stronger capacity to activate LPA receptors than the *trans*-AzoLPA form, induced by 460 nm light (54). We treated cultured DRG neurons with AzoLPA pre-exposed to the respective light wavelengths. Our results showed that neither form of AzoLPA promoted axon elongation (*SI Appendix*, Fig. S2*D*), suggesting that LPA receptor activation alone is insufficient to enhance axon growth. These findings imply that PA and LPA promote axon regeneration primarily through intracellular signaling pathways rather than through extracellular receptors. Overall, our study elucidates that lipin1 coordinates mTOR and STAT3 activity by modulating the cellular abundance of PA and LPA.

### Reciprocal Regulation between mTOR and Lipin1 in RGCs.

**Fig. 3.**
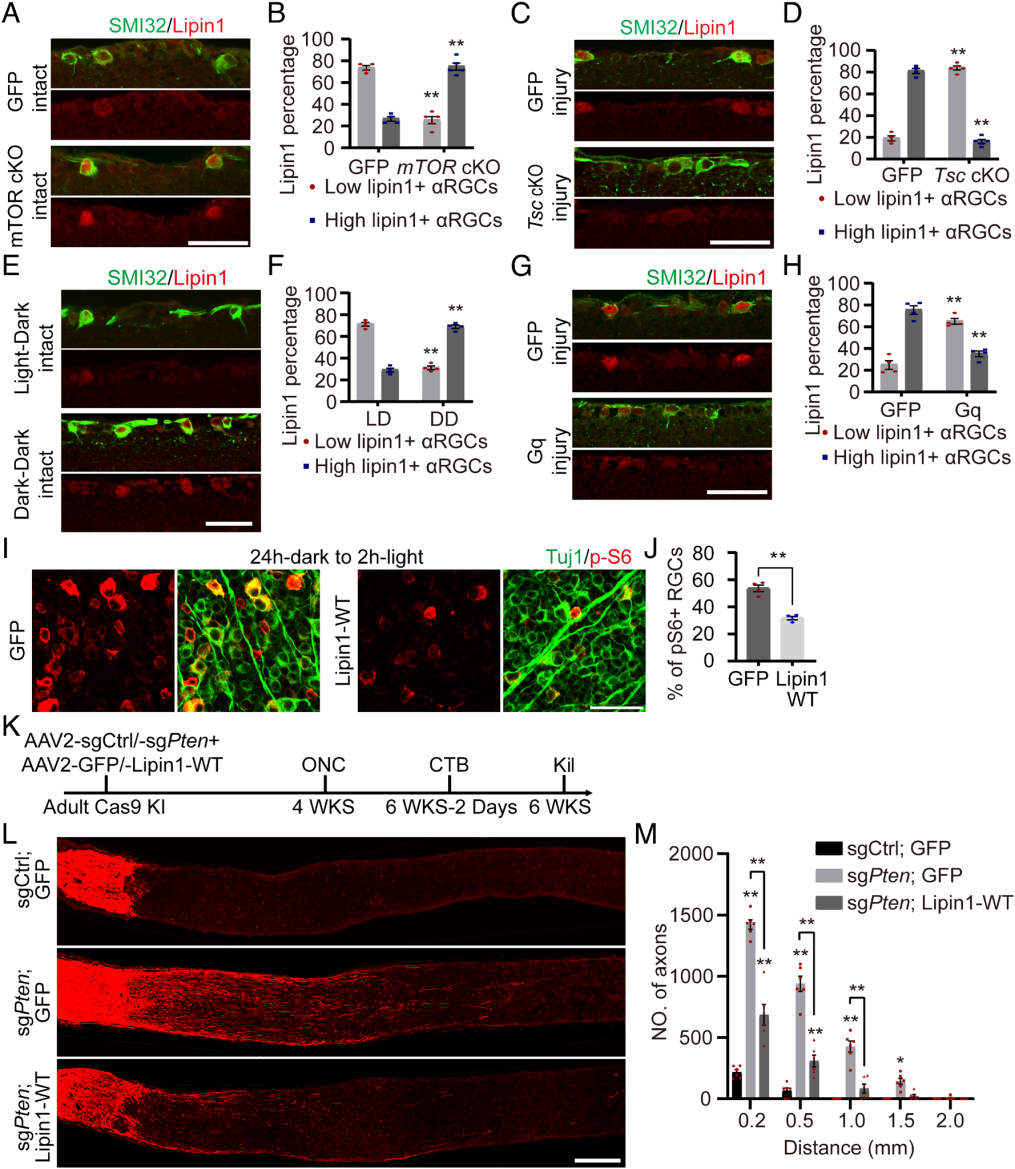
Reciprocal control of lipin1 and mTOR signaling regulates axon regeneration after optic nerve injury. (*A* and *B*) Lipin1 expression in the retinal sections from *mTOR*^f/f^ mice with AAV2-Cre or AAV2-GFP injection (*A*) and quantification (*B*). (Scale bar: 50 μm.) **P ≤ 0.01, ANOVA followed by Šídák’s test, n = 4 to 5 mice. (*C* and *D*) Lipin1 expression in the retinal sections from *Tsc*^f/f^ mice with AAV2-Cre or AAV2-GFP injection (*C*) and quantification (*D*). (Scale bar: 50 μm.) **P ≤ 0.01, ANOVA followed by Šídák’s test, n = 4 to 5 mice. (*E* and *F*) Lipin1 expression in the retinal sections from WT mice under normal light:dark circadian or with 1 d dark:dark adaption (*E*) and quantification (*F*). (Scale bar: 50 μm.) **P ≤ 0.01, ANOVA followed by Šídák’s test, n = 4 mice. (*G* and *H*) Lipin1 expression in the retinal sections from WT mice with indicated treatments (*G*) and quantification (*H*). (Scale bar: 50 μm.) **P ≤ 0.01, ANOVA followed by Šídák’s test, n = 4 mice. (*I* and *J*) Phospho-S6 levels in the retinas from WT mice with AAV2-GFP or AAV2-Lipin1-WT injection (*I*) and quantification (*J*). (Scale bar: 50 μm.) **P ≤ 0.01, Student’s *t* test, n = 4 mice. (*K*) Experiment schematic. (*L* and *M*) Sections of optic nerves with indicated treatments (*L*) and quantification (*M*). (Scale bar: 200 μm.) **P ≤ 0.01, *P ≤ 0.05, ANOVA followed by Tukey’s test, n = 6 mice. See also *SI Appendix*, Fig. S3.

Following injury, mTOR activity decreases significantly and remains at a low level (27). Previously, we showed that lipin1 expression increases 3 d after injury (4), which is later than mTOR inactivation. This observation prompted us to investigate the interaction between mTOR and lipin1. First, we examined whether mTOR could regulate lipin1 expression. We used *mTOR*^f/f^ mice and delivered AAV2-GFP or AAV2-Cre into the eyes. Retina sections were immunostained with SMI32 and lipin1 antibodies to evaluate lipin1 expression in the αRGCs, the primary cell type regenerating axons after lipin1 depletion. We observed that mTOR cKO resulted in more αRGCs expressing high-level lipin1 compared with the WT αRGCs (Fig. 3 *A* and *B*), suggesting that diminished mTOR activity leads to the upregulation of lipin1. Following optic nerve injury, mTOR activity decreases while lipin1 expression increases (4, 26). Next, we investigated whether boosting mTOR activity reverses this process. Tuberous sclerosis complex (TSC) inhibits mTOR activity by acting as a GTPase activating protein for Ras homolog enriched in the brain, a critical mTOR activator (55). TSC elimination up-regulates mTOR activity (26). We administered AAV2-Cre or AAV2-GFP to the eyes of *Tsc1*^f/f^ mice and subjected them to optic nerve injury. Three days after injury, most αRGCs expressed high-level lipin1 in the GFP group, while the majority of RGCs displayed low lipin1 expression following *Tsc1* cKO (Fig. 3 *C* and *D*). These findings collectively indicate that mTOR negatively influences lipin1 expression.

It has been shown that neuronal activity can impact mTOR signaling (27). To examine the mTOR–lipin1 interaction under physiological conditions, we investigated whether lipin1 expression was influenced by neuronal activity. We collected retinas from rodents exposed to either a 12 h:12 h light:dark cycle or a 1-d adaptation to a 12 h:12 h dark:dark cycle. Consistent with previous reports (27), dark adaption decreased the c-Fos and p-S6 levels in the RGCs (*SI Appendix*, Fig. S3 *A* and *B*). The αRGCs with dark adaptation showed higher lipin1 expression compared with those under normal light condition (Fig. 3 *E* and *F*). These findings indicate that inhibiting neuronal activity elevates lipin1 expression. Next, we asked whether enhancing neuronal activity could reduce the high-level lipin1 expression in the injured αRGCs. We used the Designer Receptors Exclusively Activated by Designer Drugs (DREADD) system to boost neuronal activity. We injected AAV2-GFP or AAV2-DREADD-Gq into the eyes of wild-type (WT) mice and allowed 4 wk for viral expression. Subsequently, we conducted optic nerve injury and administered intraperitoneal injections of the designer drug Clozapine N-oxide (CNO) (5 mg/kg) once daily. After 3 d of treatment, the mice received a final dose of CNO and were killed 2 h later. Notably, the αRGCs with DREADD-Gq stimulation expressed lower-level lipin1 compared with the control αRGCs (Fig. 3 *G* and *H*). The DREADD-Gq stimulation was verified by increased c-Fos and p-S6 expression in the RGCs (*SI Appendix*, Fig. S3 *C* and *D*). These results indicate that mTOR activation, through enhancing neuronal activity, down-regulates lipin1 expression. Altogether, our results suggest a negative regulatory role of mTOR in lipin1 expression.

Our previous data demonstrate that mTOR suppresses lipin1 expression and lipin1 KD elevates mTOR activity. Oppositely, we asked whether lipin1 overexpression is sufficient to inhibit mTOR activity. We delivered AAV2-Lipin1-WT into the eyes of adult WT mice to overexpress human WT lipin1 (lipin1-WT) (4). Four weeks later, animals were adapted to a dark environment for 24 h, followed by room light stimulation for 2 h. Under this context, the p-S6 level was high in the control group. In contrast, p-S6 expression dramatically decreased after lipin1 overexpression (Fig. 3 *I* and *J*). Lipin1 overexpression was confirmed by lipin1 staining (*SI Appendix*, Fig. S3*E*). These data indicate that elevating lipin1 expression suppresses mTOR activity. Taken together, our data reveal that lipin1 functions as a positive feedback inhibitor of mTOR in the RGCs.

As mTOR regulates lipin1 expression and lipin1 overexpression suppresses mTOR activity, we next investigated whether Lipin1 expression played a role in the axon regeneration induced by the activation of the mTOR signaling pathway. We utilized the CRISPR-Cas9 system to conditionally delete the negative regulator of the mTOR signaling pathway, *Pten*. Cas9 knock-in (KI) mice were injected with AAV2-sgCtrl or AAV2-sg*Pten* mixed with AAV2-GFP or AAV2-Lipin1-WT. Four weeks later, the rodents received an optic nerve injury, and the optic nerve was traced with CTB 2 d before the rodents were killed (Fig. 3*K*). After *Pten* cKO, RGCs expressed lipin1 at a low level, while lipin1-WT overexpression significantly increased lipin1 expression (*SI Appendix*, Fig. S3*F*). *Pten* cKO coupled with lipin1-WT overexpression resulted in significantly less and shorter axon regeneration compared with *Pten* cKO only (Fig. 3 *L* and *M*). *Pten* cKO, combined with lipin1-WT or GFP expression, induced higher RGC survival than the Ctrl (*SI Appendix*, Fig. S3 *G* and *H*). Together, our data demonstrate that mTOR modulates Lipin1 expression to determine axon regeneration. In summary, our findings establish an mTOR–lipin1-PA/LPA–mTOR loop that represents feedback signaling to suppress mTOR activity in injury neurons continuously and consequently impedes axon regeneration.

### Lipin1 KD Facilitates CST Sprouting after Unilateral Pyramidotomy.

Given that mTOR and STAT3 signaling pathways are known to influence the regeneration capacity of CST (25, 40), we investigated whether lipin1 plays a role in the CST regrowth process. First, we examined whether lipin1 KD activated mTOR and STAT3. We delivered AAV9-shCtrl or AAV9-shLipin1 into the cortex of neonatal WT mice and collected cortex samples when the mice reached adulthood. We used RNAscope in situ hybridization to detect lipin1 mRNA in cortical neurons. The cells infected with AAV9-shLipin1 had lower lipin1 mRNA levels than the cells infected with AAV9-shCtrl (*SI Appendix*, Fig. S4*A*), indicating a successful lipin1 KD. Interestingly, neurons with lipin1 KD had greater p-S6 levels than control neurons (*SI Appendix*, Fig. S4*B*). However, p-STAT3 was not activated in either the control or lipin1 KD groups (*SI Appendix*, Fig. S4*C*). These results indicate that lipin1 KD enhances mTOR signaling but not STAT3 signaling in cortical neurons.

**Fig. 4.**
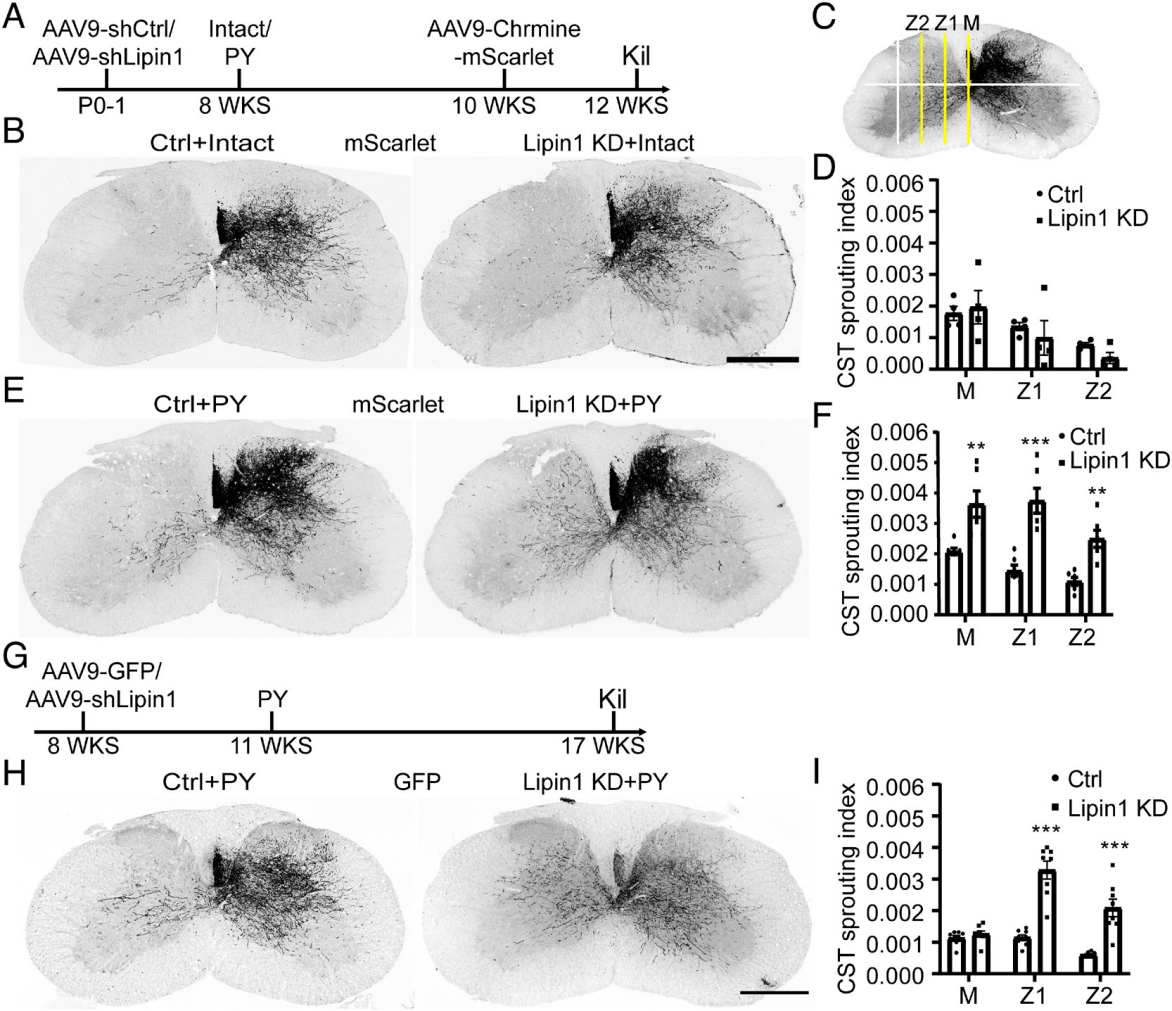
Lipin1 KD promotes CST sprouting after unilateral pyramidotomy. (*A*) Experiment schematic. PY, pyramidotomy. (*B*) Representative coronal sections of C7 spinal cord from intact ctrl and lipin1 KD mice. mScarlet indicates the CST axons. (Scale bar: 500 μm.) (*C*) Quantification paradigm. The cervical spinal cord was divided into different zones. Axons crossing the midline (M), Z1, and Z2 were quantified. (*D*) Quantification. Two-way ANOVA followed by Sidak’s multiple comparison test. n = 4 mice. (*E* and *F*) Representative coronal sections of C7 spinal cord from ctrl and lipin1 KD mice with PY (*E*) and quantification (*F*). (Scale bar: 500 μm.) Two-way ANOVA followed by Sidak’s multiple comparison test. **P ≤ 0.01, ***P ≤ 0.001. n = 6 mice. (*G*) Experiment schematic. (*H* and *I*) Coronal sections of the C7 spinal cord with indicated treatments (*H*) and quantification (*I*). (Scale bar: 500 μm.) Two-way ANOVA followed by Sidak’s multiple comparison test. ***P ≤ 0.001. n = 8 mice. See also *SI Appendix*, Fig. S4.

Then, we assessed whether lipin1 KD enhanced CST sprouting following unilateral pyramidotomy. Neonatal WT mice were injected with AAV9-shCtrl or AAV9-shLipin1 into the cortex. Eight weeks later, the mice were kept intact or underwent unilateral pyramidotomy. Two weeks after the surgery, AAV9-ChRmine-mScarlet was injected into the cortex to trace the CST through the expression of red fluorescent mScarlet. Four weeks after the pyramidotomy, the animals were killed, and their spinal cords were collected to analyze CST sprouting (Fig. 4*A*). Without pyramidotomy, the Ctrl and lipin1 KD showed similar basal CST sprouting to the contralateral side (Fig. 4*B*). To quantify the sprouting CST axons, the gray matter was evenly divided to three zones. The axons crossing the midline (M), Z1, and Z2 were quantified, and normalized with the total CST number quantified at the pyramid (Fig. 4*C*). There was no significant difference between the Ctrl and Lipin1 KD group (Fig. 4*D*). After pyramidotomy, a few CST axons sprouted to the contralateral side in the Ctrl group, whereas the lipin1-KD animal exhibited extensive sprouting to the contralateral side (Fig. 4 *E* and *F*). These findings demonstrate that neonatal lipin1 KD promotes CST sprouting after pyramidotomy.

Furthermore, we investigated whether modulation of lipin1 expression in adulthood could reverse regeneration failure. Adult WT mice received injections of AAV9-GFP or AAV9-shLipin1 mixed with AAV9-GFP, followed by the pyramidotomy 3 wk later. The experiment terminated 6 wk after the pyramidotomy (Fig. 4*G*). CST (GFP+) sprouting in the cervical spinal cord was then analyzed. Interestingly, lipin1 KD in adult mice also resulted in significantly stronger CST sprouting than control (Fig. 4 *H* and *I*). This result suggests that inhibiting lipin1 in adulthood enhances CST sprouting, implying strong potential for clinical translation.

### Lipin1 KD Promotes CST Regeneration after Juvenile SCI.

Next, we determined the effect of lipin1 KD to enhance CST regeneration following SCI. We started with the axon regeneration of juvenile animals when the animals maintain some regeneration capacity. We did T8 SCI on postnatal day 7 (P7), P14, and in adult mice, and labeled CST by cortical infection of AAV9-GFP. Consistent with previous studies (56, 57), robust CST fiber regeneration occurred after P7 SCI, but regeneration failed after adult SCI (*SI Appendix*, Fig. S5). Interestingly, after SCI at P14, most CST axons terminated at the rostral margin of the lesion site, with only a few axons growing across the lesion site (*SI Appendix*, Fig. S5). Additionally, as indicated by GFAP immunostaining, the astrocytic scar resembled that observed after adult SCI (*SI Appendix*, Fig. S5). Thus, P14 SCI presents a potential model to assess the promotional effect of novel candidates.

**Fig. 5.**
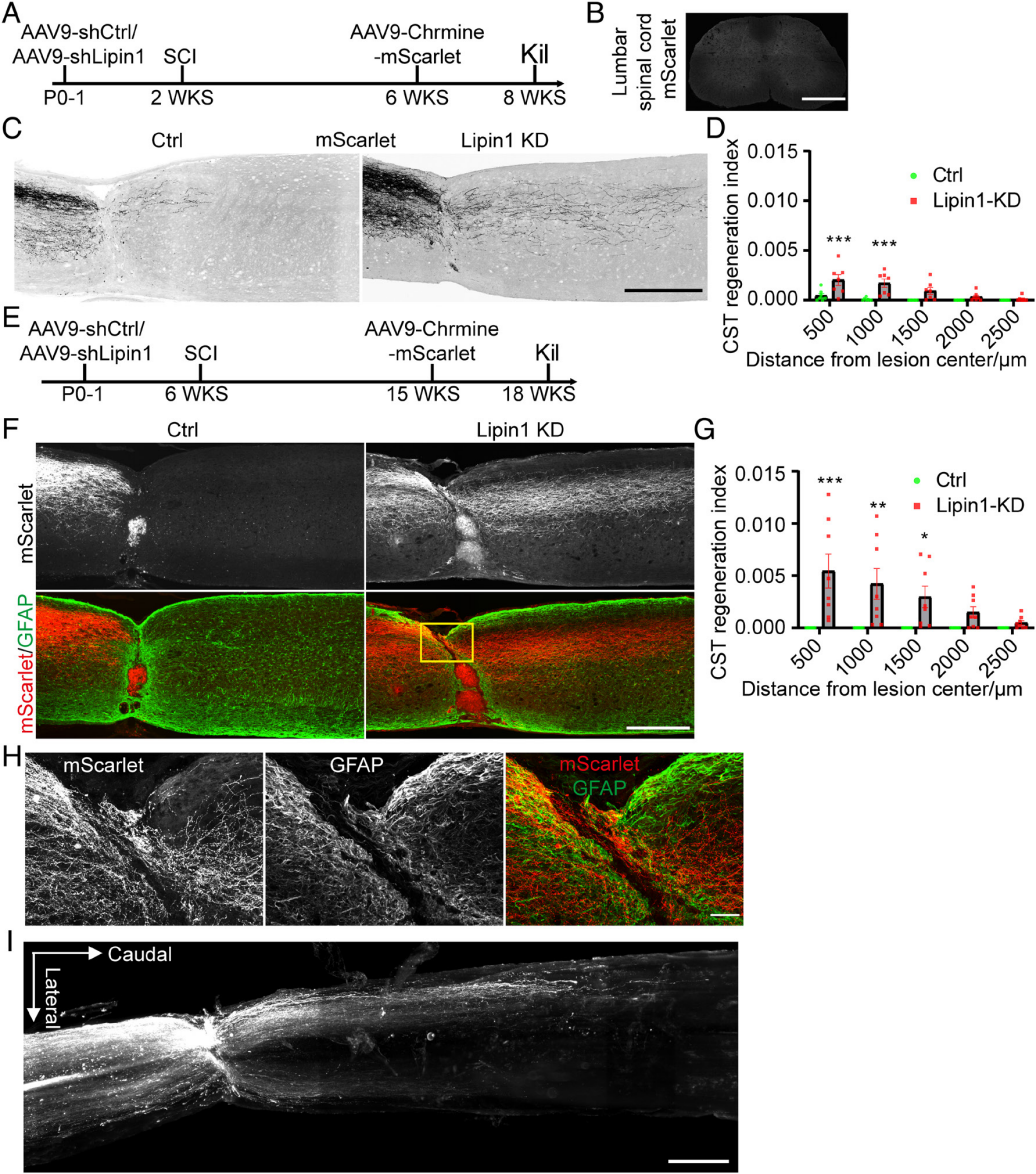
Lipin1 KD enhances CST regeneration after T8 spinal cord crush. (*A*) Experiment schematic. (*B*) mScarlet signal at the lumbar spinal cord caudal to the lesion site. (Scale bar: 500 μm.) (*C* and *D*) Sagittal spinal cord sections from ctrl and lipin1 KD animals (*C*) and quantification (*D*). mScarlet indicated the regenerating CST axons. (Scale bar: 500 μm.) Two-way ANOVA followed by Sidak’s multiple comparison test. **P ≤ 0.01, ***P ≤ 0.001. n = 7 mice. (*E*) Experiment schematic. (*F* and *G*) CST axons regeneration of Ctrl and lipin1 KD mice (*F*) and quantification (*G*). Immunostaining of mScarlet and GFAP was performed to visualize CST axons and astrocytic scar. (Scale bar: 500 μm.) Two-way ANOVA followed by Sidak’s multiple comparison test. *P ≤ 0.05, ***P ≤ 0.001. n = 8 mice. (*H*) Zoom in images of the boxed area in (*F*). (*I*) Orthogonal projection image of the spinal cord with tissue clearing and 3D imaging. (Scale bar: 500 μm.) See also *SI* *Appendix*, Figs. S5–S9 and Movies S1–S6.

We next examined the effect of lipin1 KD on CST regeneration using the P14 SCI model. Neonatal WT mice received AAV9-shCtrl or AAV9-shLipin1 injections into the cortex and were subjected to a complete spinal cord crush at P14. Four weeks later, the CST was traced using a cortical injection of AAV9-ChRmine-mScarlet. After 6 wk, the animals were killed (Fig. 5*A*). The injury completeness was confirmed by the absence of mScarlet-positive fibers at the distal lumbar spinal cord (Fig. 5*B*). In the control group, only a few regrowing axons were observed, terminating approximately 500 μm caudal to the lesion site (Fig. 5 *C* and *D*). In contrast, the lipin1-KD animals exhibited a significantly larger number of regenerated axons, with the longest axons innervating the spinal segment 2,000 μm caudal to the lesion site (Fig. 5 *C* and *D*). These findings demonstrate that lipin1 KD promotes CST axon regeneration in young rodents.

### Lipin1 KD Promotes Robust CST Regeneration after Complete Adult SCI.

The promising results of axon regeneration induced by lipin1 KD in juvenile rodents motivated us to investigate its effects on adult CST regeneration. We injected AAV9-shCtrl or AAV9-shLipin1 into the cortex of neonatal WT mice and performed a complete T8 SCI at 6 wk. The animals received CST tracing using a cortical injection of AAV9-ChRmine-mScarlet at 15 wk and were killed at 18 wk (Fig. 5*E*). In the control group, CST axons (mScarlet+) terminated at the proximal margin of the astrocytic scar (Fig. 5 *F* and *G*). In contrast, abundant CST axons regrew across the lesion site and innervated up to 3 mm caudal the lesion site (Fig. 5 *F* and *G*). Consistent with the previous report (58), the regenerated axons were associated with GFAP+ cells in the lesion center (Fig. 5*H*). Additionally, we presented all the control mice and lipin1-KD mice to demonstrate typical CST axon regeneration (*SI Appendix*, Figs. S6 and S7). To confirm the completeness of the SCI, the absence of mScarlet and 5-HT in the distal spinal cord of all control and lipin1-KD animals was confirmed (*SI Appendix*, Figs. S6 and S7). Moreover, to unambiguously assess the CST regeneration following lipin1 KD, we conducted tissue clearing and 3D imaging of two spinal cord segments collected 4 mo post adult SCI. The cortical neurons with AAV9-shLipin1 infection expressed GFP and subsequently traced the axons. Numerous CST axons were visualized in the transparent spinal cord (Fig. 5*I*). The lesion site was identified by disturbance of axon trajectory (Fig. 5*I*). A large amount of CST axons regenerated into the lesion site. The longest axons of animal 1 extended 3 to 4 mm and animal 2 extended 2.5 mm caudal to the lesion site (Fig. 5*I*, *SI Appendix*, Fig. S8, and Movies S1–S6). Collectively, our data demonstrate that neonatal lipin1 KD significantly promotes CST axon regeneration following complete SCI in adult mice.

Next, we examined CST regeneration after adult lipin1 KD. Adult WT mice were administered AAV9-GFP or AAV9-shLipin1 into the cortex. The complete T8 SCI was applied 2 wk later. The animals were killed 14 wk post SCI (*SI Appendix*, Fig. S9*A*). The AAVs expressed GFP, which tracked the CST axons. Some CST axons regrew across the lesion site (*SI Appendix*, Fig. S9 *B* and *C*). We verified the injury completeness by the loss of GFP and 5HT signal in the lumbar spinal cord (*SI Appendix*, Fig. S9*D*). These data suggest that lipin1 KD at the adult stage promotes CST axon regeneration. Taken together, we demonstrate that lipin1 is a critical suppressor of CST regeneration.

### Lipin1 KD in DRG Neurons Promotes Ascending Sensory Axon Regeneration.

**Fig. 6.**
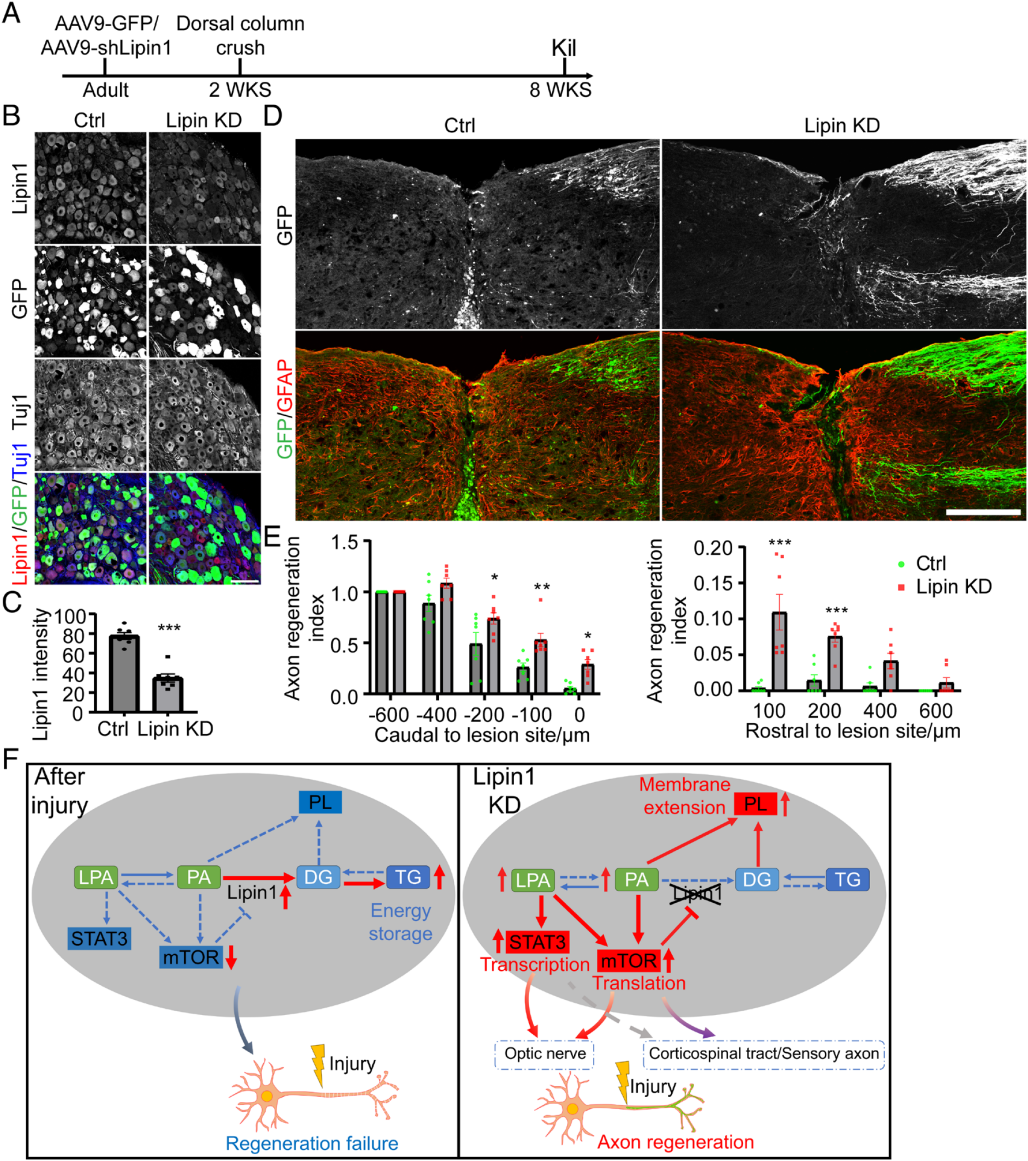
Lipin1 KD facilitates ascending sensory axon regeneration. (*A*) Experiment schematic. (*B* and *C*) DRG sections from WT mice with AAV9-GFP or AAV9-shLipin1 infection (*B*) and quantification of lipin1 expression (*C*). Immunostaining of lipin1 (red) and Tuj1 (blue) was performed. (Scale bar: 100 μm.) Students *t* test. ***P ≤ 0.001. n = 7. (*D* and *E*) Regeneration of ascending sensory axons with indicated treatment (*D*) and quantification (*E*). GFP expression driven by the AAVs was used to indicate regenerating axons. Immunostaining GFP (green) and GFAP (red) was performed to visualize the sensory axons and astrocytic scar. (Scale bar: 200 μm.) Two-way ANOVA followed by Sidak’s multiple comparison test. *P ≤ 0.05, **P ≤ 0.01, ***P ≤ 0.001. n = 7 mice. (*F*) Proposed mechanism for axon regeneration after lipin1 KD. See also *SI Appendix*, Fig. S10.

We previously reported that lipin1 KD elevates DRG neurite outgrowth in vitro (4). However, the role of lipin1 in regulating sensory axon regeneration after SCI has remained unclear. To address this, we injected AAV9-GFP or AAV9-shLipin1 into the DRGs of adult WT mice. We performed a dorsal column crush to transect the ascending sensory axons completely 2 wk later and killed all the mice 6 wk postinjury (Fig. 6*A*). DRG sections were immunostained for lipin1 and Tuj1 to confirm the lipin1 KD. GFP expression indicated a successful AAV infection (Fig. 6*B*). DRG neurons infected with AAV9-shLipin1 showed 55% less lipin1 expression than those with AAV9-GFP infection (Fig. 6 *B* and *C*), indicating successful lipin1 KD. Lipin1 KD induced a higher p-S6 level than the Ctrl (*SI Appendix*, Fig. S10*A*). However, the p-STAT3 levels were similar in the two groups (*SI Appendix*, Fig. S10*B*). Next, we evaluated the regeneration of ascending sensory axons. In the control animals, the ascending sensory axons halted at the proximal margin of the lesion site, with only a few axons regenerating into the lesion site (Fig. 6 *D* and *E*). In contrast, many axons regenerated into the lesion center and grew into the rostral side in the lipin1 KD group (Fig. 6 *D* and *E*). Overall, our data suggest that lipin1 plays an important role in the regeneration capacity of ascending sensory axons.

## Discussion

Lipin1 serves a dual role within the cell, functioning as a PAP governing glycerolipid metabolism and a transcription coactivator upon translocation to the nucleus (59, 60). Early studies revealed that mTORC1 directly phosphorylates lipin1, thereby regulating its cellular localization (61). However, our previous study demonstrated that the nucleus location of lipin1 was not essential to axon regeneration (4). Our current study showed that lipin1 concurrently modulated mTOR and STAT3 signaling pathways to determine axon regeneration (Fig. 6*F*). Following lipin1 KD, mTOR and STAT3 were activated in the RGCs. Both mTOR and STAT3 were required for lipin1 KD–induced optic nerve regeneration. The potential mediators are PA and LPA. The PA is known to activate mTOR activity through direct binding (11). We showed that neuronal PA level increased after lipin1 KD. Cellular delivery of PA was sufficient to activate mTOR and promote axon regeneration. As to LPA, it can be synthesized from PA and is also up-regulated after lipin1 KD (4, 9). It is heavily documented that LPA functions as an extracellular signal and interacts with at least six G protein-coupled receptors (GPCRs) to mediate diverse cell behaviors, including cell proliferation, survival, and migration (62). However, the intracellular function of LPA is not well described. Our data evidenced that intracellular delivery of LPA boosted mTOR and STAT3, promoting substantial neurite outgrowth. Interestingly, lipin1 KD also promotes CST and sensory axon regeneration. However, only mTOR was activated in the cortical and DRG neurons, indicating diverse cellular lipid metabolism and signaling across different neuron types. Collectively, our study demonstrates that lipin1 orchestrates cell signaling pathways by modulating the cellular abundance of PA and LPA, which together with reorganized PLs synthesis determine the regeneration capacity of CNS axons.

After CNS injury, mTOR activity tends to decrease and remain low, accompanied by limited axon regeneration (26–28). Reactivation of mTOR long after injury promotes axon regeneration, suggesting that mTOR suppression acts as a persistent barrier to regeneration (28). Nevertheless, how the low mTOR activity is sustained in the injured CNS neurons remains unclear. In this study, we provided several lines of evidence to show that mTOR tightly suppressed lipin1 expression. Traumatic injury to the optic nerve decreases mTOR activity in the RGCs (26), leading to upregulation of lipin1. Depleting lipin1 restored mTOR activity. Oppositely, lipin1 overexpression down-regulated mTOR activity in the intact RGCs. This feedback signaling is likely mediated by PA and LPA, as discussed previously. Altogether, we unveiled that mTOR-lipin1-PA/LPA-mTOR serves as a feedback loop to maintain low mTOR activity in the CNS neurons and subsequently suppresses the regeneration ability.

The CST is a direct connection between cortical neurons and the spinal cord, crucial in transmitting essential information for fine motor control (63). However, the limited regenerative capacity of the CST poses a significant obstacle to functional recovery following SCI. Although substantial progress has been made in our understanding of CNS axon regeneration in recent decades, it has been challenging to effectively promote robust axon regeneration after SCI, particularly in cases of complete SCI (64). This study found that lipin1 KD enhances CST and sensory axon regeneration, suggesting that it plays conserved roles in CNS and PNS neurons. However, whether the regenerated axons reform neural networks and restore function remains to be investigated. A recent study revealed that chemoattracting and guiding the axons of Vsx2+ neurons to reinnervate their natural target regions resulted in significant walking recovery after complete SCI (65). This warrants further studies to examine whether suppressing lipin1 expression in these neurons could promote axon regeneration of these spinal neurons. Furthermore, it is important to consider that axon guidance factors may also be necessary for other regenerated axons to establish functional connections. Taken together, our findings present lipin1 as a potential therapeutic target for restoring axon regeneration after SCI.

## Materials and Methods

### Materials.

Materials and reagents used in this study are provided in *SI Appendix*, Tables S1–S3.

### Animals.

All experimental procedures were performed in compliance with animal protocols approved by the Laboratory Animal Facility at the Hong Kong University of Science and Technology. *Stat3*^f/f^ mice were gifts from Zhenguo Wu (Hong Kong University of Science and Technology, Hong Kong, China). Rosa26-Cas9, *mTOR*^f/f^, *Rptor*^f/f^, and *Tsc1*^f/f^ mice were purchased from the Jackson Laboratory. *Rptor*^f/f^ mice were crossed with *Stat3*^f/f^ mice to generate the double KO mice. Only female mice were used in the complete SCI experiments.

### AAV Preparation.

AAVs were packaged into distinct serotypes to meet the needs of different neural tissues. AAV serotype 2/2 was used for GFP, Cre, or shRNA expression in RGCs. AAV serotype 2/9 was employed to express GFP, Chrmine-mScarlet, or shRNA in the cortex and DRG. The virus titer was measured by qPCR, and the AAVs were titrated to 2*10^12^-1*10^13^ virus genocopies (GC)/mL before being used in the experiments.

### Surgeries.

For surgical procedures involving neonatal or P7 mice, mice were anesthetized with hypothermia induced by ice embedding. For the other surgeries, mice received a mixture of ketamine (80 mg/kg) and xylazine (10 mg/kg) for anesthesia. After surgery, mice were placed on the heating pad to maintain their body temperature until they were completely awake. Ketoprofen (0.05 mL/kg) was administered as an analgesic after the surgical operation.

Details on experimental procedures used in this study, including optic nerve injury, pyramidotomy, SCI, immunohistochemistry, cell culture, WB, lipidomics, and statistics can be found in *SI Appendix*, *SI Methods*.

## Supplementary Material

Appendix 01 (PDF)

Movie S1. Video showing continuous spinal cord slices with regenerated axons from ventral to dorsal side for animal 1.

Movie S2. 3D reconstruction of light-sheet microscope-imaged spinal cord rotating via X axis for animal 1. The X, Y, and Z axes point to the caudal, lateral, and dorsal spinal cords, respectively.

Movie S3. Spinal cord rotating via Y axis for animal 1.

Movie S4. Spinal cord rotating via Z axis for animal 1.

Movie S5. Zoom in perspective for animal 1.

Movie S6. Video showing continuous spinal cord slices with regenerated axons from ventral to dorsal side for animal 2.

## Data Availability

All study data are included in the article and/or supporting information.
