# Supplementary material for: Lipin1 depletion coordinates neuronal signaling pathways to promote motor and sensory axon regeneration after spinal cord injury
Source: Proc Natl Acad Sci U S A. 2024 Sep 18;121(39):e2404395121. doi: 10.1073/pnas.2404395121 (PMC11441493; doi:10.1073/pnas.2404395121)
Supplement: Supplementary file 1 — Appendix 01 (PDF) [file pnas.2404395121.sapp.pdf]

PNAS

Supporting Information for

Lipin1 depletion coordinates neuronal signaling pathways to promote motor and sensory axon regeneration after spinal cord injury

Weitao Chen[a,1], Junqiang Wu[b,1], Chao Yang[b,c,d,1], Suying Li[e,f,g,h], Zhewei Liu[b], Yongyan An[b], Xuejie Wang[b,c], Jiaming Cao[b], Jiahui Xu[b,c,d], Yangyang Duan[b,c,d], Xue Yuan[c], Xin Zhang[b], Yiren Zhou[b], Jacque Pak Kan Ip[i], Amy K. Y. Fu[b,c,d], Nancy Y. Ip[b,c,d], Zhongping Yao[e,f,g,h], Kai Liu[a,b,c,d,j,*]

[a]Biomedical Research Institute, Shenzhen Peking University–The Hong Kong University of Science and Technology Medical Center, Shenzhen 518036, China
[b]Division of Life Science, State Key Laboratory of Molecular Neuroscience, The Hong Kong University of Science and Technology, Hong Kong, China
[c]Hong Kong Center for Neurodegenerative Diseases, Hong Kong, China
[d]Guangdong Provincial Key Laboratory of Brain Science, Disease and Drug Development; Hong Kong University of Science and Technology Shenzhen Research Institute; Shenzhen-Hong Kong Institute of Brain Science, Shenzhen, Guangdong 518057, China
[e]State Key Laboratory of Chemical Biology and Drug Discovery, Research Institute for Future Food, Research Centre for Chinese Medicine Innovation, The Hong Kong Polytechnic University, Hung Hom, Kowloon, Hong Kong Special Administrative Region, China
[f]Department of Applied Biology and Chemical Technology, The Hong Kong Polytechnic University, Hung Hom, Kowloon, Hong Kong Special Administrative Region, China
[g]State Key Laboratory of Chinese Medicine and Molecular Pharmacology (Incubation), Hong Kong Polytechnic University Shenzhen Research Institute, Shenzhen 518057, China
[h]Shenzhen Key Laboratory of Food Biological Safety Control, Hong Kong Polytechnic University Shenzhen Research Institute, Shenzhen 518057, China
[i]School of Biomedical Sciences, The Chinese University of Hong Kong, Hong Kong SAR, China
[j]Department of Chemical and Biological Engineering, The Hong Kong University of Science and Technology, Hong Kong, China

[1]W.C, J.W and C.Y contributed equally to this work.

*Corresponding author: Kai Liu.

Email: kailiu@ust.hk

This PDF file includes:

- SI Methods
- Figures S1 to S10
- Tables S1 to S3
- Legends for Movies S1 to S6
- SI References

Other supporting materials for this manuscript include the following:

- Movies S1 to S6

SI Methods

Optic nerve injury. The intravitreal injection and optic nerve lesion were carried out as described previously (1). Briefly, the conjunctiva was exposed by clamping the eyelid with an artery clamp. Two microliters of vitreous medium were extracted using a Hamilton syringe, and 2 μL of AAV was gently infused. Two to four weeks after the AAV ($2x10^{12}$-$1x10^{13}$ GC/mL) injection, the optic nerve was exposed and then compressed for two seconds with #5 Dumont forceps. Twelve days after the injury, 2 μL of CTB (1 mg/mL) was injected into the eye to label regenerating axons.

To quantify the axon regeneration, the optic nerve was cryosectioned longitudinally at a thickness of 8 μm (t=8 μm). The CTB signal was amplified by immunostaining. Five sections of each animal were imaged with a confocal microscope. The optic nerve width (D) and the number of regenerating axons (n) were measured at various distances from the lesion site. The maximum D (Dm) measured was deemed the optic nerve's diameter. The total number of regenerating axons was calculated using the formula: $N = \pi \times (Dm/2)^2 \times (\text{average } n/D)/t$.

To quantify the survival of RGCs, retinas with whole-mount staining of Tuj1 were divided into four quadrants. Using a confocal microscope with a 63X objective, three images were captured from the periphery to the center in each quadrant. To measure lipin1, p-S6, p-AKT473, p-ERK, and p-STAT3 expression, respective antibodies were used to stain retinal cryosections. At least ten images were taken with a confocal microscope for each animal. For lipin1 quantification, the mean intensity of lipin1 in the αRGCs and background was measured with ImageJ. RGCs with fluorescence intensity ten times higher than the background intensity are classified as high lipin1 expression. For p-S6, p-AKT473, and p-ERK quantification, the RGCs exhibiting staining intensity higher than 5 folds of the background were determined as positive RGCs. For p-STAT3 quantification, RGCs with nucleus aggregation of bright p-STAT3 signal were considered positive cells (2). The positive rates were determined by the number of RGCs with respective positive signals dividing the number of Tuj1 positive RGCs.

Neonatal cortical injection. The cortical injection for the neonatal mice was performed as previously described (3). Briefly, 2 μL of AAVs ($5x10^{12}$ GC/mL) were injected into two sites of the sensorimotor cortex using a microforged glass pipette adapted for the nanoject II (Drummond) device. After injection, the neonates were placed on a heating pad until they were fully awake and returned to their home cage.

Adult cortical injection. Cortical injection was performed as described previously (3). Briefly, animals were immobilized on a stereotaxic apparatus. An incision along the midline was performed to expose the skull, followed by a craniotomy to expose the sensory and motor cortex. For pyramidotomy experiments, AAVs ($2x10^{12}$ GC/mL) were injected at two sites: 0.6 mm deep, 1.5

mm lateral, and 0 and 1 mm anterior to bregma. For the SCI experiment, AAVs ($2x10^{12}$ GC/mL) were injected at two sites: 0.6 mm in deep, 1.5 mm lateral, and 1 and 0 mm posterior to bregma. Each site received 500 nL of AAV at a rate of 100 nL/min. The wound was sutured after injection.

Pyramidotomy. Pyramidotomy was performed as described previously (3). Briefly, mice under anesthesia were placed ventral side up. Over the trachea, a midline incision was made. The skull base was exposed by carefully dissecting the covering tissue. Through a craniotomy, the medullary pyramid was exposed. Then, the unilateral pyramid was transected with a feather microscalpel (Electron Microscopy Sciences). After the pyramidotomy, the skin was sutured.

The C7 spinal cord was coronally sectioned and imaged with a confocal microscope to analyze the CST sprouting quantitatively. The completeness of pyramidotomy was confirmed by immunostaining of Phosphokinase C γ (PKCγ). The CST axons were indicated by mScarlet. A horizontal line was drawn from the central canal to the rim of the gray matter, and then three vertical lines were drawn at the median and trisection points. The number of axons crossing the vertical lines was counted, and the average number of CST branches was obtained by measuring three sections of each mouse. The total number of CST axons was quantified at the medullary pyramid from two sections. The CST sprouting index represents the ratio of the number of sprouting CST axons to the total number of CST axons.

Spinal cord injury. T8 spinal cord crush procedures were performed as previously described (3). Briefly, the skin over the thoracic vertebrae was incised. A laminectomy was conducted to expose the T8 spinal cord. Then, the spinal cord was crushed for 2 seconds with a refined #5 jeweler's forceps. After surgery, the wound was sutured layer by layer. The rodents were warmed with a heating pad until fully awake, after which they were returned to their home cage. Throughout the period with the SCI, manual urine expression was performed twice daily. For the P7 and P14 spinal cord injuries, pups were coated with feces from their mother before being returned to their mother.

To quantify CST regeneration, sagittal sections were imaged with a confocal microscope. Vertical lines were drawn at different distances from the lesion center. CST axons intersecting the vertical lines were quantified in three sections per animal. The average number of regenerating axons per section was then normalized using the total CST number measured at the pyramid. The injury completeness was validated by 5HT+ and mScarlet+ axon elimination at the lumbar spinal cord. Animals with spared axons or failed AAV infection were excluded.

To measure the neuronal p-S6 and p-STAT3 expression, the cortex samples were sectioned at 10 μm thickness and immunostained with respective antibodies. The samples were imaged with the confocal microscope. For the intensity of p-S6, more than 60 GFP+ pyramidal neurons from

each animal were measured by ImageJ. For the p-STAT3, the percentage of p-STAT3 positive neurons in the GFP+ neurons was quantified.

DRG injection. Adult WT mice were anesthetized and positioned under a surgical microscope. A midline incision was made in the lumbar region. A laminectomy was performed to expose two of the L3-5 DRGs. Using a pulled glass needle adapted with a Hamilton syringe, 1 μL of AAV was smoothly administered into each DRG. Finally, the surgical wound was sutured in layers.

Dorsal column crush. Dorsal column crush was performed as previously described (4). In brief, a midline incision was made to expose the thoracic vertebrae, and the T8 spinal cord was subsequently exposed. The dorsal column at T8 was crushed using modified #5 jeweler's forceps at a depth of 0.8 mm. After six weeks, the animals were killed, and both the spinal cord and DRGs were collected for further analysis.

For the measurement of lipin1, p-S6 and p-STAT3 expression, the DRGs were sectioned at 10 μm thickness and stained with respective antibodies. The samples were imaged using a confocal microscope. The mean intensity of lipin1 and p-S6 in at least 40 GFP+ DRG neurons was quantified using ImageJ for each mouse. The percentage of p-STAT3 positive DRG neurons was quantified in three sections of each animal.

To quantify sensory axon regeneration, three sagittal sections of each animal were imaged using a confocal microscope. Vertical lines were drawn at different distances from the lesion center. The crossing axons were quantified and normalized to the number of axons at 600 μm caudal to the lesion center.

Immunohistochemistry. Mice received an overdose of ketamine/xylazine to be deeply anesthetized. Then, transcardial perfusion was performed using ice-cold PBS, followed by 4% PFA in PBS. Neural tissues, including the retina, optic nerve, brain, and spinal cord, were dissected and subsequently postfixed in 4% PFA overnight.

For whole-mount staining, the retina was rinsed with PBS three times to remove residual PFA and then incubated with 4% normal goat serum and 0.1% Triton-100 in PBS (NGST) for 30 minutes. The retina was then incubated overnight with the primary antibodies in NGST, followed by PBS washing for three times and the secondary antibodies incubation for 2 hours. Finally, the retinas were rinsed with PBS to remove unbound secondary antibodies and mounted onto glass slides.

To prepare samples for cryosectioning and immunostaining, neural tissues were cryoprotected with 30% sucrose for two days and subsequently embedded in the optimum cutting temperature (OCT, Tissue-Tek) medium. The embedded tissues were then sectioned and mounted on glass slides. After washing with PBS to remove residual OCT, the samples were blocked with NGST for

30 minutes. Then, the slides were incubated with primary antibodies in NGST. After the overnight incubation, the slides were washed six times, followed by incubation with the corresponding secondary antibodies. Two hours later, the secondary antibodies were washed three times, and the slides were mounted with a cover glass. Heating-mediated antigen retrieval in sodium citrate buffer was performed before staining lipin1, p-AKT473, p-Stat3, and p-ERK.

RNAscope in situ hybridization. Lipin1 mRNA in situ hybridization was done on the cortex section (10 μm) following the user manual of the RNAscopeTM Multiplex Fluorescent Reagent Kit v2(323100). GFP staining was performed after RNA hybridization. Images were taken under Zeiss LSM 980 confocal microscope(20x). The RNA puncta(dots) in individual GFP+ cells were counted.

PA and LPA preparation. DP-PA, PO-PA, P-LPA, and O-LPA (Larodan) were encapsulated within lipid vesicles using a previously established protocol (5). Briefly, PAs and LPAs were reconstituted in a vesicle buffer consisting of 150 mM NaCl and 10 mM Tris-Cl (pH 8.0). The lipid suspension was subsequently sonicated to generate small vesicles suitable for cellular uptake. The freshly prepared lipid vesicles were then introduced into the culture medium.

DRG neuron culture. Primary culture of DRG neurons and replating culture were performed as previously described (1, 4). In brief, L3-L5 dorsal root ganglia (DRGs) were dissected from adult WT mice and digested in 0.5% collagenase for 90 minutes. Subsequently, the digestion medium was replaced with a culture medium (Neurobasal-A supplemented with penicillin-streptomycin and B-27). The digested DRGs were then triturated with a pipette 20-30 times to isolate DRG neurons. The isolated DRG neurons were plated onto a 6-well plate pre-coated with Poly-D-Lysine and laminin. For WB analysis, the DRG neurons were cultured for four days before being collected for protein extraction. Lipid vesicles containing PAs and LPAs (500μM) were added to the medium 30 minutes before cell collection. For replating culture, DRG neurons were cultured for five days, and PAs and LPAs (10μM) in the lipid vesicles were added to the medium daily, from day 1 to day 4. The cells were then detached from the plate by pipetting 20-30 times and replated onto a 24-well plate. The replating culture was terminated with 4% PFA after 18-20 hours. Tuj1 staining was performed to visualize DRG cell bodies and neurites. The length of the longest neurite was quantified using ImageJ.

For DRG culture with LPA receptor antagonists, 1μM TAK615 (LPA_1 antagonist) (6), 10μM Ki16425 (LPA_1 and LPA_3 antagonist) (7) and 1μM AM095 (LPA_1 antagonist) (8) was applied together with PBS or LPA in the lipid vesicle.

For DRG culture with AzoLPA, AzoLPA (Avanti) was first dissolved in DMSO at 5 mM and then irradiated by either 460 nm blue light or 365 nm UV-A light for 1 min to obtain *trans*-AzoLPA or *cis*-

AzoLPA, respectively (9). Each of them was added to DRG cell cultures at a final concentration of 10 μM. All steps were protected from room light. DMSO vehicles irradiated at the same wavelengths were used as controls.

Primary cortical neuron culture. Primary cortical neuron cultures were established using cortical neurons isolated from embryonic day 18 (E18) mice. Briefly, brains were dissected in ice-cold Hanks' balanced salt solution (HBSS, Gibco) and then digested with 2.5% Trypsin (Gibco) in HBSS supplemented with Ca^{2+}, Mg^{2+}, and Hepes/HCl for 20 minutes. Subsequently, the digestion medium was replaced with 10% fetal bovine serum (FBS, Cytiva) to inactivate trypsin. Before cell seeding, the culture plate was coated with poly-d-lysine. The isolated cells were cultured in a neurobasal medium (Gibco) supplemented with B27 (Gibco), 1% penicillin-streptomycin (Gibco), and GlutaMax (Gibco). On day 1 in vitro (DIV 1), AAV-scramble or AAV-shLipin1 was added to the culture medium. To inhibit glial proliferation from DIV 1 to DIV 7, 5Fdu (2.5μM, Sigma) was applied. After one week of culture, the cells were harvested for lipidomic analysis.

Western Blots. Cultured DRG neurons were harvested and lysed in RIPA buffer for 45 minutes. The RIPA buffer consisted of 50 mM Tris-HCl at pH 8.0, 150 mM NaCl, 1% Nonidet P-40, 0.5% Na-deoxycholate, and 0.5% SDS. Prior to cell lysis, EDTA-free complete ULTRA tablets (Roche) and PhosSTOP Complete Easypack (Roche) were added to the RIPA buffer. The lysed sample was then centrifuged at 16,000g for 10 minutes, and the resulting supernatant was combined with 5x SDS sample buffer [300 mM Tris-HCl buffer, 10% SDS, 5% beta-mercaptoethanol, 50% glycerol, and 0.05% bromophenol blue]. The mixture was heated to 100 °C for 15 minutes. Western Blots was performed following the standard protocol.

Lipid extraction and UPLC-MS. Lipid extraction and UPLC-MS were performed as previously described (1). Using the Folch method, a 2:1 mixture of chloroform and methanol was applied to rupture the cultured cortical neurons. The upper phase was collected, and the solvent was dried by line blowing with nitrogen. For UPLC-ESI-MS analysis, an Orbitrap Exploris™ 480 mass spectrometer coupled with an Ultimate 3000 UPLC system was employed. Separation on a Hypersil GOLD™ C18 column (particle size: 1.9 mm; length: 100 mm; i.d.: 2.1 mm) was performed at a column temperature of 50 °C. The mobile phase consisted of solvent A (water: acetonitrile, 40:60, v/v) and solvent B (isopropanol: acetonitrile, 90:10, v/v), both containing ten mM ammonium acetate and 0.1% formic acid. The mobile phase was pumped at a flow rate of 0.3 mL/min, and the following gradient elution program was applied: 0-2 min, 40% B; 2-2.5 min, 40%-58% B; 2.5-18 min, 58%-99% B; 18-25 min, 99% B; 25-25.1 min, 99%-40% B; 25.1-29 min, 40% B.

MS data acquisition encompassed positive and negative ion modes, with a scan range from 100 to 1500 Da. The capillary voltage settings were adjusted to 3.5 kV and 2.5 kV for positive and negative ion modes, respectively. The ESI source parameters were configured as follows: ion transfer tube temperature at 300 °C, vaporizer temperature at 350 °C, sheath gas flow set to 30 Arb, and aux gas flow set to 10 Arb.

The acquired raw UPLC-ESI-MS data were directly imported into LipidSearch 4.2 software for subsequent steps, including peak picking, alignment, and compound identification. Semi-quantification of individual lipid species was conducted by referencing spiked internal standards obtained from Avanti Polar Lipids, namely PC (16:0-d31/18:1) and TG (16:0/18:0/16:0-d5). To identify potential biomarkers, the processed data matrices were imported into MetaboAnalyst 5.0 software. Various statistical analyses were employed, including t-tests ($p < 0.05$), fold change (threshold 1.5), and OPLS-DA (VIP > 1), to select and characterize potential biomarkers.

Tissue clearing and imaging. The protocol for tissue clearing was modified from iDISCO+(10, 11) and Adipo-Clear(12) protocols. Briefly, four months after the SCI, the spinal cord was dissected after transcardiac perfusion with PBS and 4% PFA. After washed with PBS, the spinal cord was dehydrated by methanol/B1N buffer series and delipidated by dichloromethane (DCM). A bleaching step was applied by incubating the sample in methanol/H_2O_2 mixture, followed by rehydration with methanol/B1N buffer series. The sample was washed in PTxWH buffer and then went through immunostaining with primary antibody and secondary antibody (Alexa Fluro 647 conjugated). After washing, the sample underwent another round of dehydration by Methanol/ddH_2O buffer series and delipidation. Then, the sample was incubated in dibenzyl ether (DBE) and was ready for imaging.

The sample was immobilized in a square-shaped holder, emersed in DBE, and imaged by a LiTone XL Light-sheet Microscope. The sample was scanned at a step of 4 μm. Stitching of raw data was facilitated by a Fiji plugin, BigStitcher(13). Video showing continuous slices of the imaged sample and its Z projection were also done using Fiji. AIVIA was used to generate 3D visualization and videos showing the sample rotating around each axis.

Quantification and statistical analyses. Statistical analyses of all quantification data were performed using GraphPad Prism 6 software (GraphPad Software, La Jolla, CA). Student's t-test was utilized to compare two groups, while ANOVA was employed to compare multiple groups, followed by post-hoc multiple analyses. Data are presented as the mean ± SEM. Statistical significance levels were denoted as *$p \leq 0.05$, **$p \leq 0.01$, and ***$p \leq 0.001$.

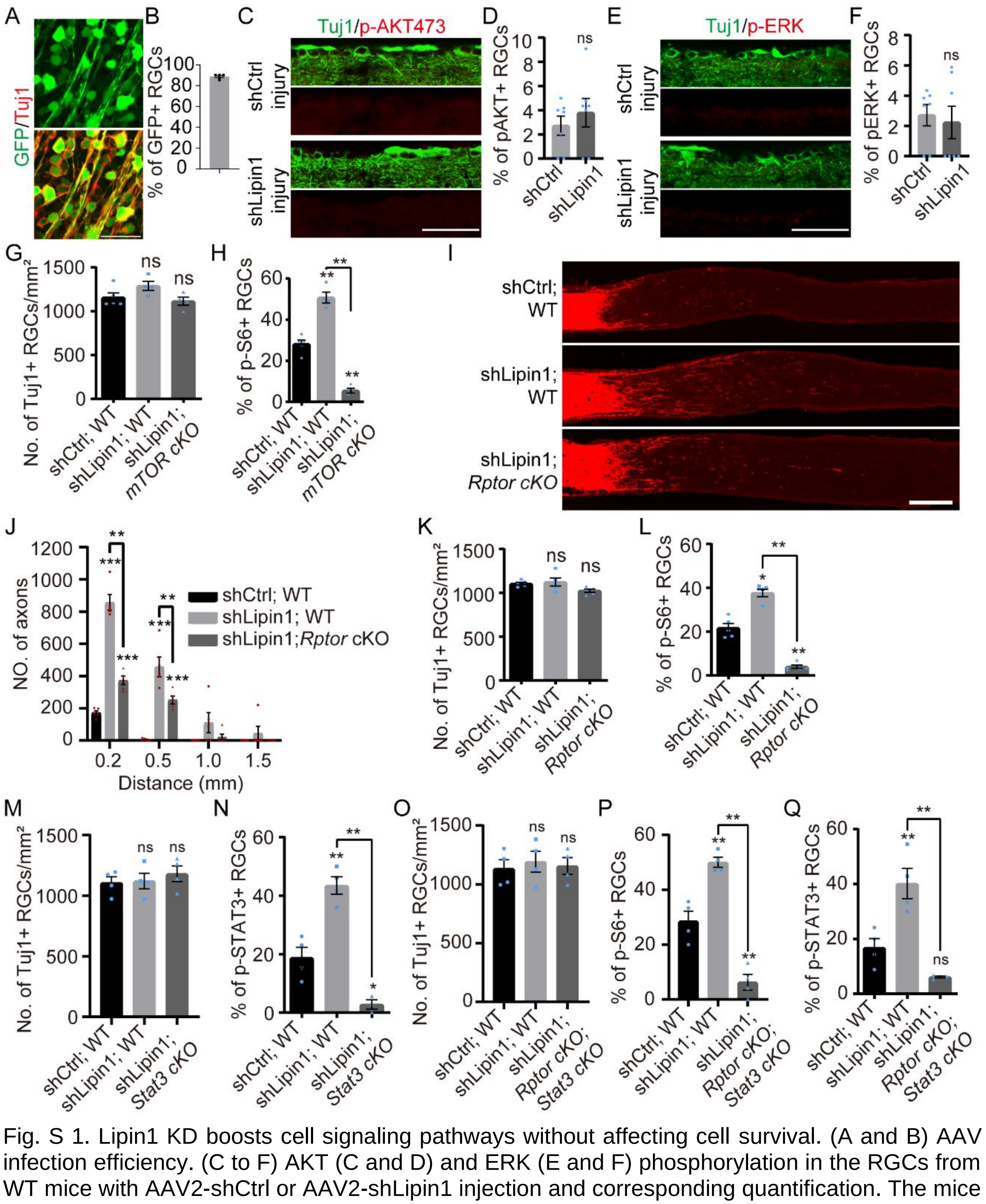

Fig. S 1. Lipin1 KD boosts cell signaling pathways without affecting cell survival. (A and B) AAV infection efficiency. (C to F) AKT (C and D) and ERK (E and F) phosphorylation in the RGCs from WT mice with AAV2-shCtrl or AAV2-shLipin1 injection and corresponding quantification. The mice received optic nerve injury at four weeks after AAV injection. The samples were collected one day after injury. Immunostaining of Tuj1 and p-AKT473 in (C) and Tuj1 and p-ERK in (D) was performed to detect protein levels. $^{**}p \leq 0.01$, ns, not significant, Student's t-test. n=7 mice. Scale bar: 50 μm. (G) Quantification of surviving RGCs at 2 weeks post injury (WPI). The samples were collected from WT or mTOR cKO mice with AAV2-shCtrl or AAV2-shLipin1 injection. ns, not significant, ANOVA followed by Tukey's test. n=4-5 mice. (H) Quantification of the percentage of p-S6+ RGCs

from respective groups at 2 WPI. **$p \leq 0.01$, ANOVA followed by Tukey's test. n=4-5 mice. (I and J) Axon regeneration with indicated treatments (I) and quantification (J). Scale bar: 200 μm. **$p \leq 0.01$, ***$p \leq 0.001$, ns, not significant, ANOVA followed by Šídák's test, n = 5 mice. (K) Quantification of surviving RGCs from respective groups at 2 WPI. ns, not significant, ANOVA followed by Tukey's test. n=5 mice. (L) Quantification of the percentage of p-S6+ RGCs from respective groups at 2 WPI. *$p \leq 0.05$, **$p \leq 0.01$, ANOVA followed by Tukey's test. n=5 mice. (M) Quantification of surviving RGCs from respective groups at 2 WPI. ns, not significant, ANOVA followed by Tukey's test. n=4 mice. (N) Quantification of the percentage of p-STAT3+ RGCs from respective groups at 2 WPI. **$p \leq 0.01$, *$p \leq 0.05$, ANOVA followed by Tukey's test. n=3-4 mice. (O) Quantification of surviving RGCs from respective groups at 2 WPI. ns, not significant, ANOVA followed by Tukey's test. n=4 mice. (P) Quantification of the percentage of p-S6+ RGCs from respective groups at 2 WPI. **$p \leq 0.01$, ANOVA followed by Tukey's test. n=4 mice. (Q) Quantification of the percentage of p-STAT3+ RGCs from respective groups at 2 WPI. **$p \leq 0.01$, ns, not significant, ANOVA followed by Tukey's test. n=4 mice.

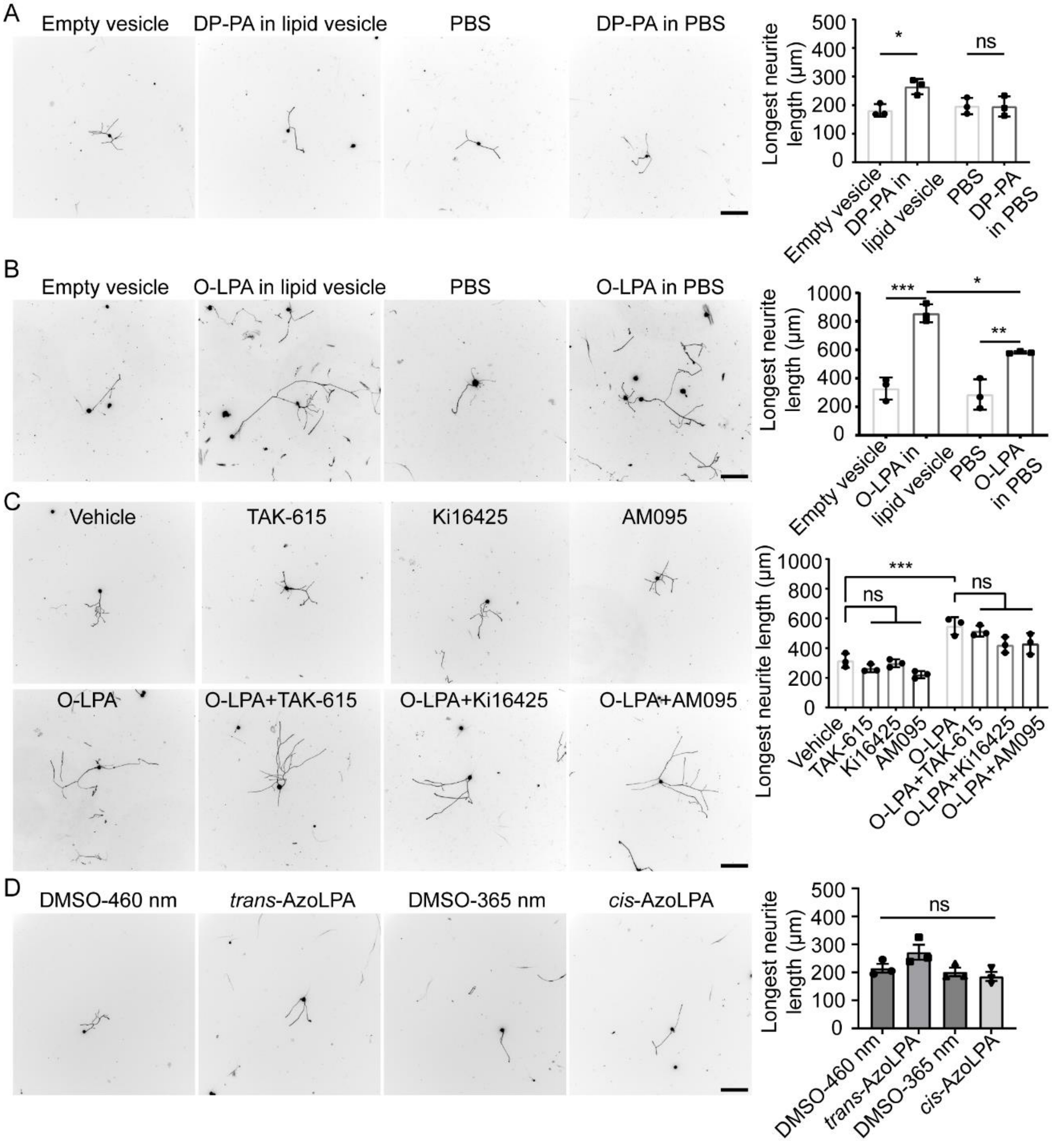

Fig. S 2. PA and LPA promote axon regeneration through intracellular signaling but not extracellular receptors. (A) DRG neurite outgrowth with lipid vesicle encapsulated PA and PBS dissolved PA, and quantification. Scale bar: 200 μm. *p≤ 0.05, ns, not significant, ANOVA followed by Sidak's test. n=3. (B) DRG culture with lipid vesicle encapsulated LPA and unencapsulated LPA. Scale bar: 200 μm. *p≤ 0.05, **p≤ 0.01, ***p≤ 0.001, ANOVA followed by Sidak's test. n=3. (C) DRG culture with LPA and LPA receptor antagonists. TAK-615, LPA_1 antagonist, 1μM; Ki16425, LPA_1 and LPA_3 antagonist, 10μM; AM095, LPA_1 antagonist, 1μM. Scale bar: 200 μm. ***p≤ 0.001, ns, not significant, ANOVA followed by Sidak's test. n=3. (D) DRG culture with AzoLPA. Scale bar: 200 μm. ns, not significant, ANOVA followed by Sidak's test. n=3.

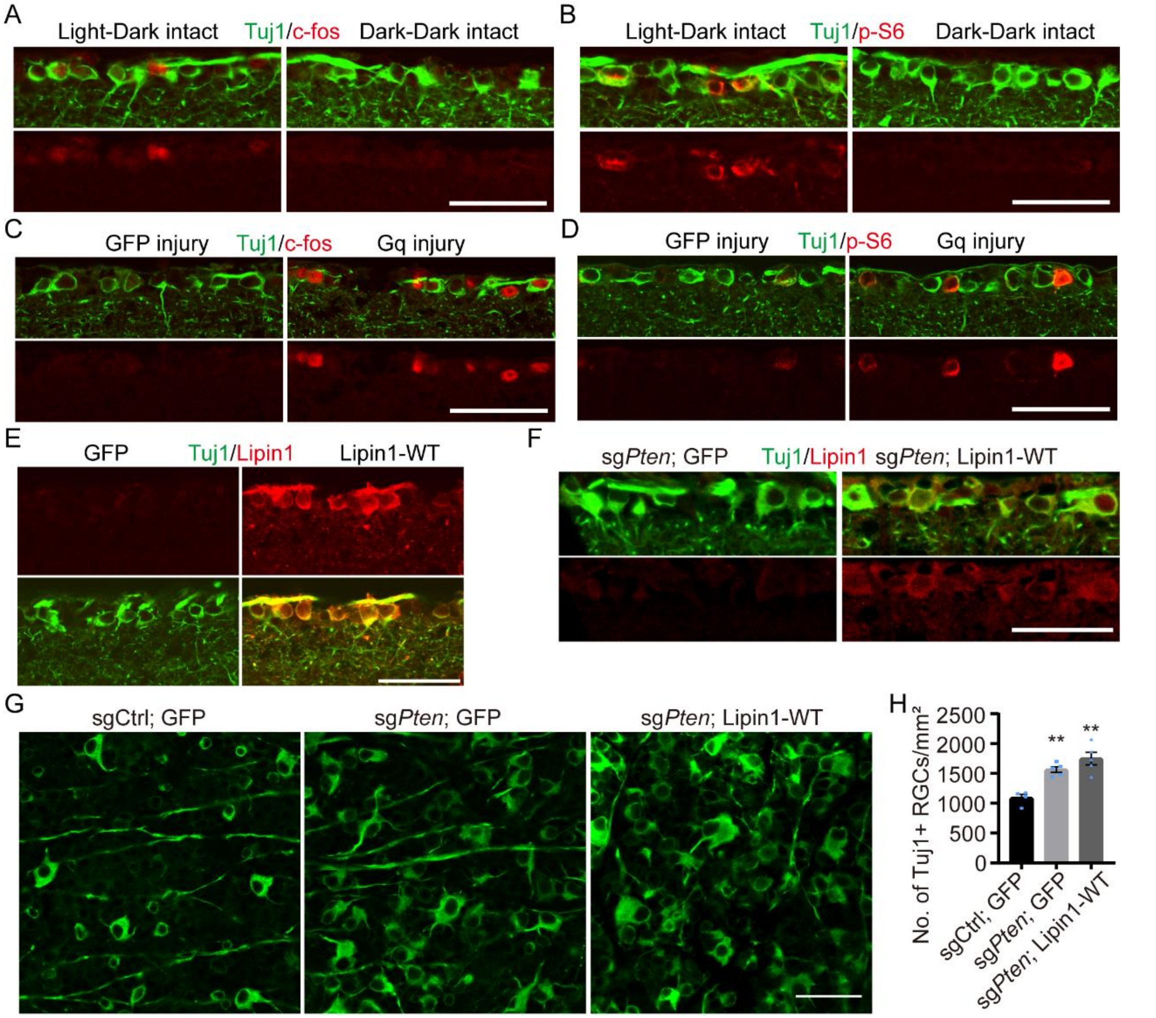

Fig. S 3. Neuronal activity regulates mTOR activity and quantification of RGC survival and pS6 expression in the RGCs. (A and B) Neuronal activity (A) and p-S6 level (B) in the RGCs from WT mice under 12h:12h light-dark circadian or with one-day dark-dark adaption. Scale bar: 50 μm. (C and D) Neuronal activity (C) and p-S6 level (D) in the RGCs from control or Gq-stimulated mice. The samples were collected three days after injury. Scale bar: 50 μm. (E) Lipin1 expression in the RGCs with AAV2-GFP or AAV2-Lipin1-WT infection. (F) Lipin1 expression in the RGCs with *Pten* cKO and lipin1 overexpression. Scale bar: 50 μm. (G) RGC survival with indicated treatments. Scale bar: 50 μm. (H) Quantification of RGC survival in (G). **p≤0.01, ANOVA followed by Tukey's test. n=4-5 mice.

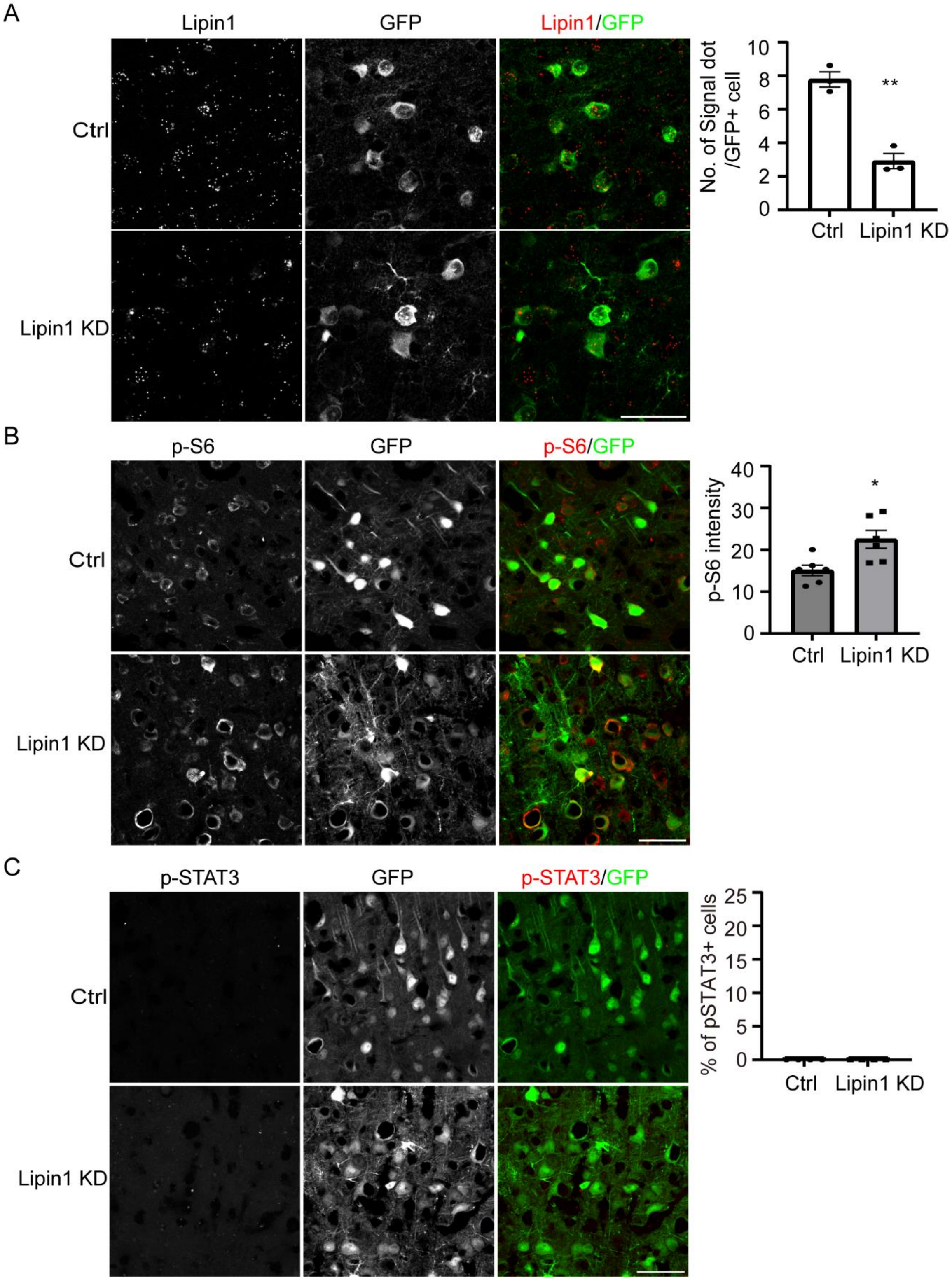

Fig. S 4. mTOR and STAT3 activation after lipin1 KD in the cortical neurons. (A) RNAscope in situ hybridization of lipin1 mRNA in the cortical neurons and quantification. **$p \leq 0.01$, Student's t-test. n=3 mice. Scale bar: 50 μm. (B) Phospho-S6 level in the cortical neurons and quantification.

*p≤0.05, Student's t-test. n=6 mice. Scale bar: 50 μm. (C) Phospho-STAT3 level in the cortical neurons and quantification. Student's t-test. n=4 mice. Scale bar: 50 μm.

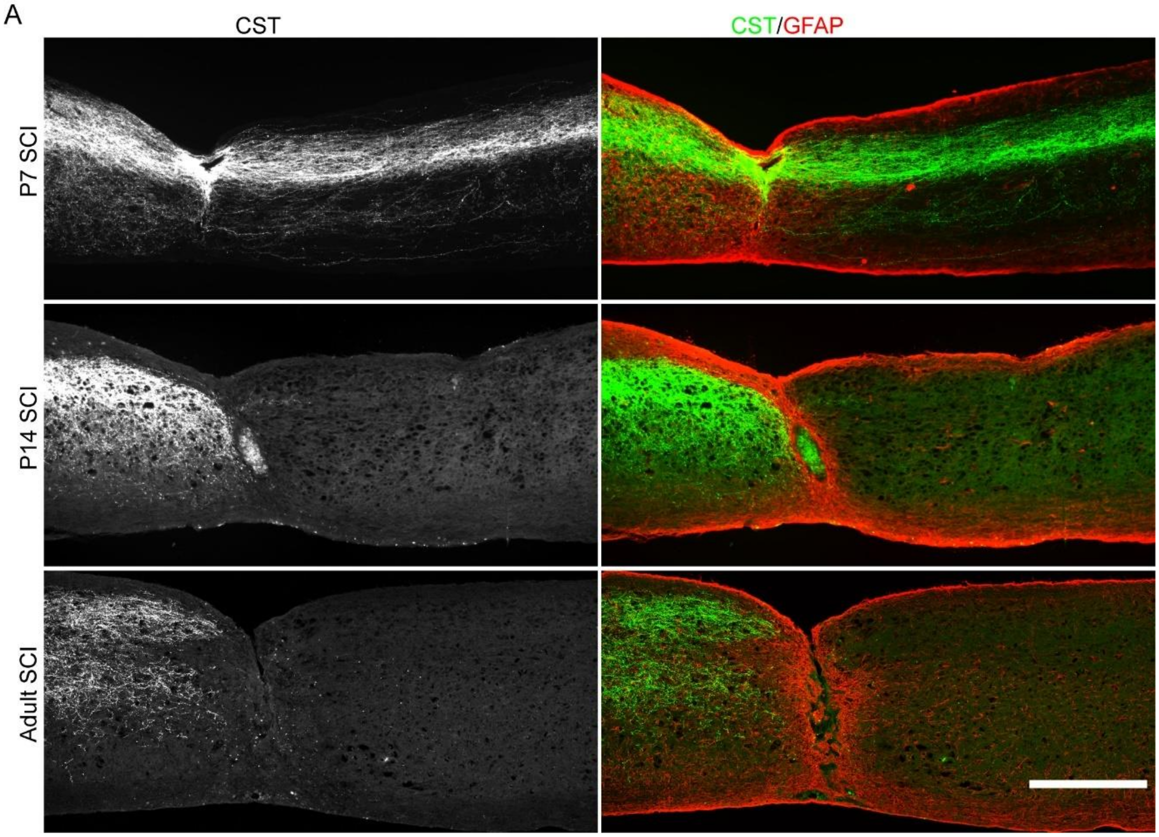

Fig. S 5. Age-dependent CST regeneration after SCI. Representative images of CST regeneration after SCI at different ages.

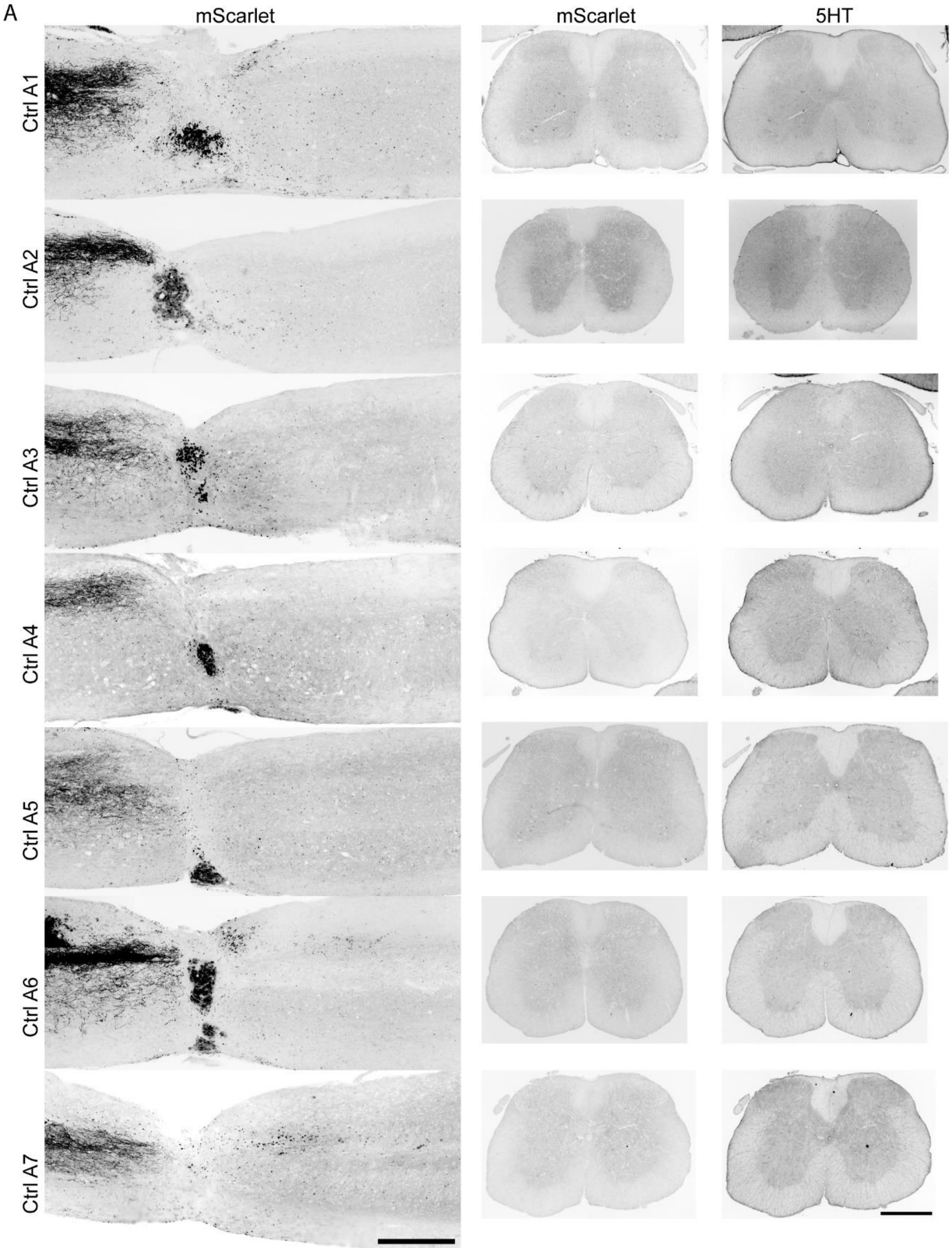

Fig. S 6. Axon regeneration of the individual Ctrl animal. The left panel displays sagittal spinal cord sections of 7 ctrl mice. The right panel shows respective coronal sections of the lumbar spinal cord immunostained with mScarlet and 5HT. Scale bar: 500µm.

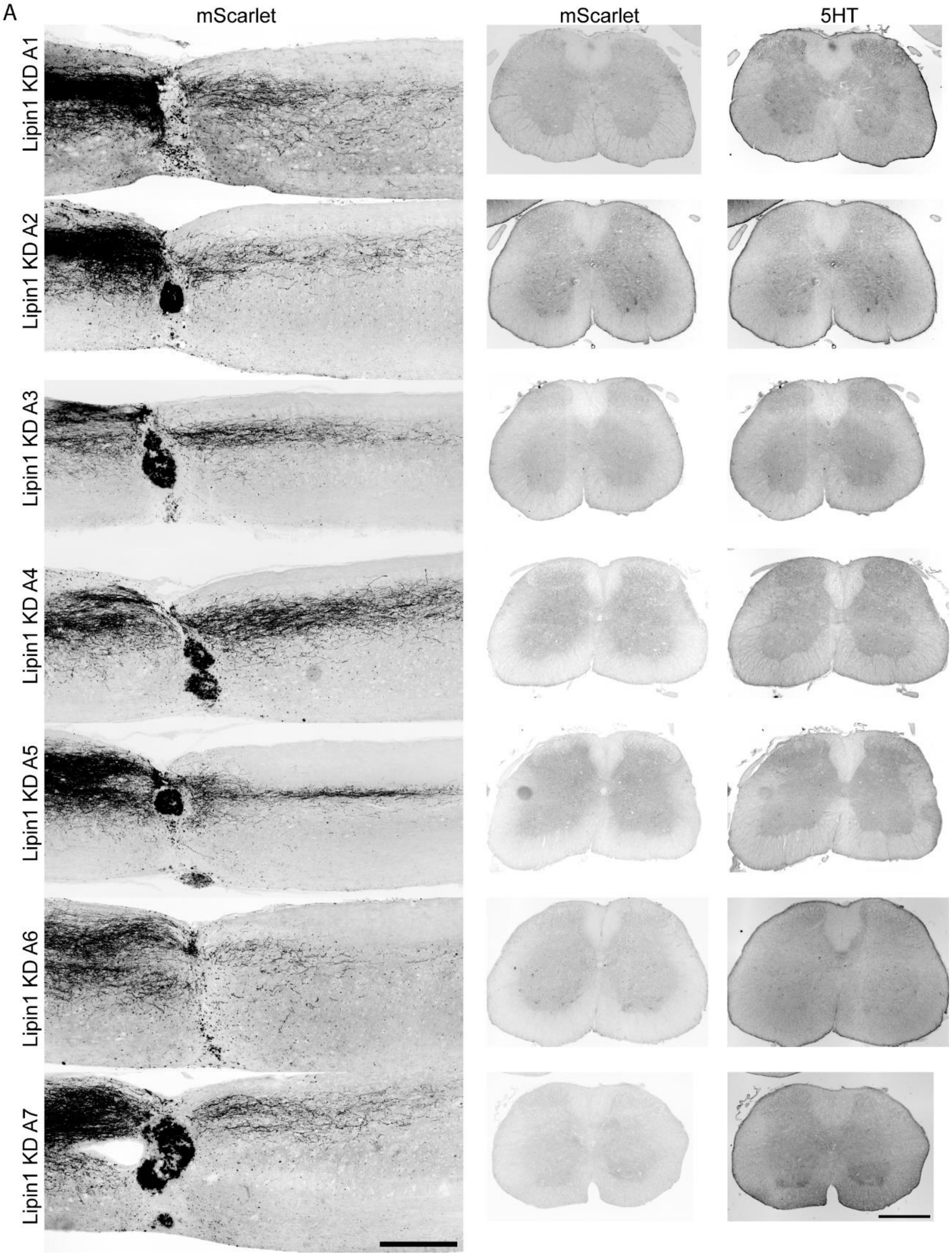

Fig. S 7. Examples of CST regeneration after lipin1 KD. The left panel displays sagittal spinal cord sections of 7 lipin1 KD mice. The right panel shows respective coronal sections of the lumbar spinal cord immunostained with mScarlet and 5HT. Scale bar: 500μm.

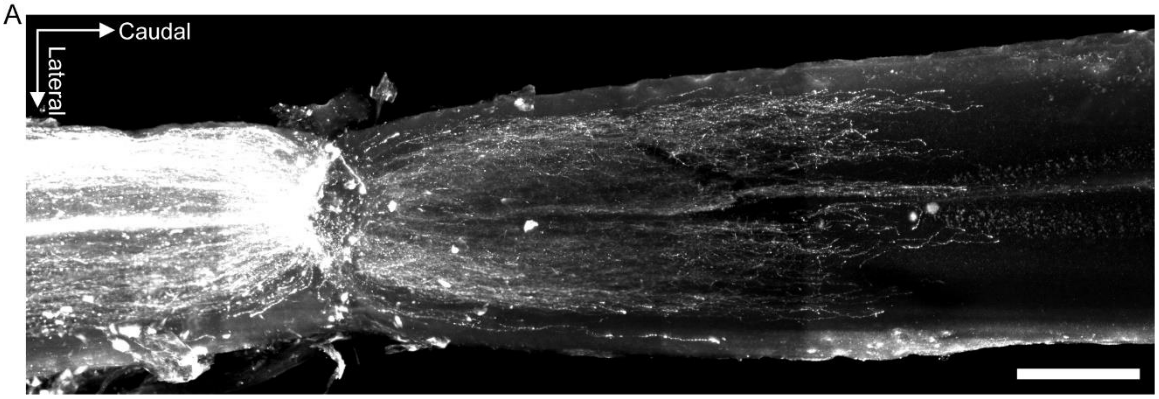

Fig. S 8. Orthogonal projection image of the spinal cord with tissue clearing and 3D imaging. Scale bar: 500μm.

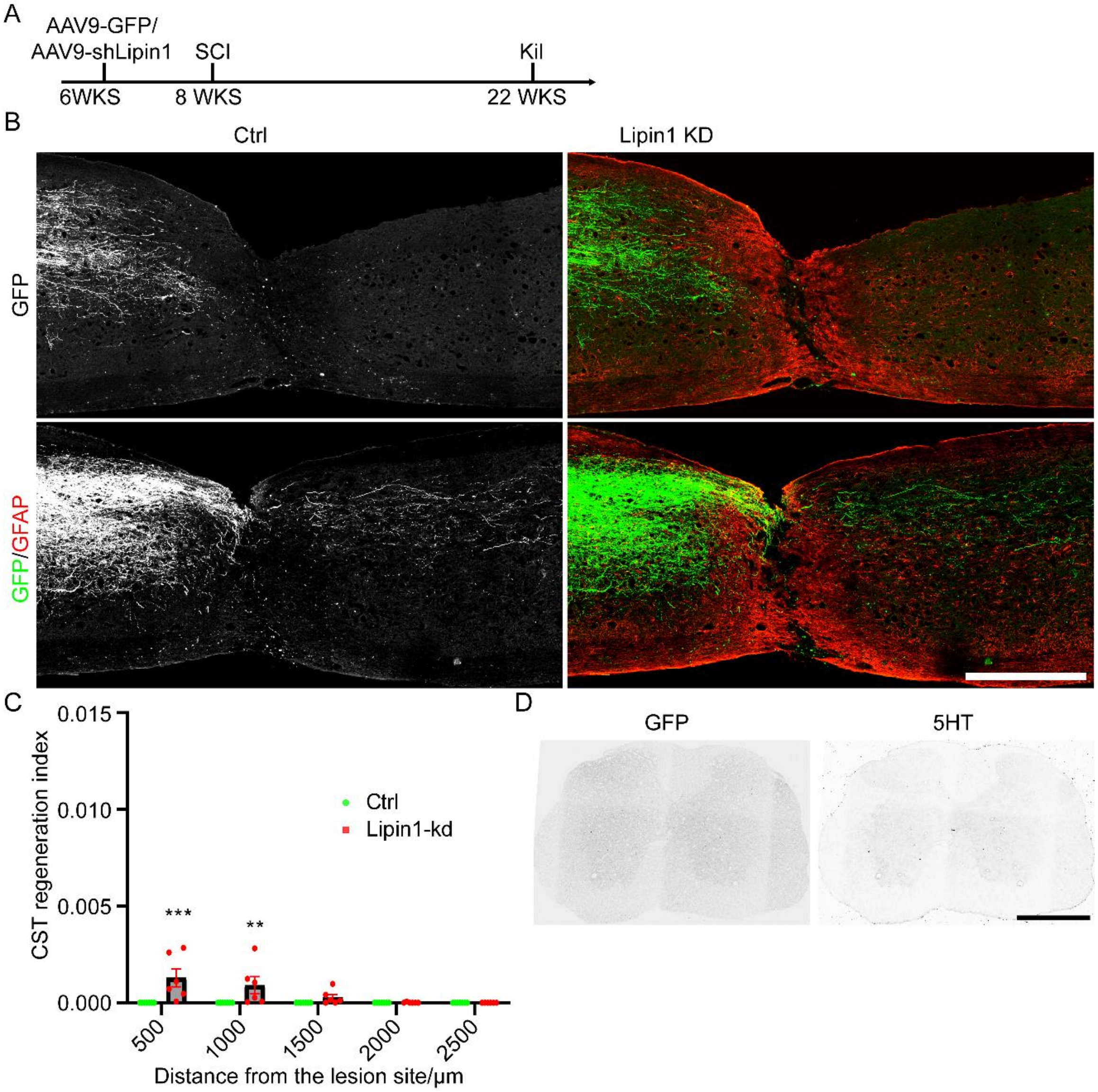

Fig. S 9. Adult lipin1 KD facilitates robust axon regeneration. (A) Experiment schematic. (B and C) CST axons regeneration of Ctrl and lipin1 KD mice (B) and quantification (C). Immunostaining of GFP and GFAP was performed to visualize CST axons and astrocytic scar. Scale bar: 500μm. Two-way ANOVA followed by Sidak's multiple comparison test. **p≤0.01, ***p≤0.001. n=6-7 mice. (D) Coronal section of lumbar spinal cord immunostained with GFP and 5HT. Scale bar: 500μm.

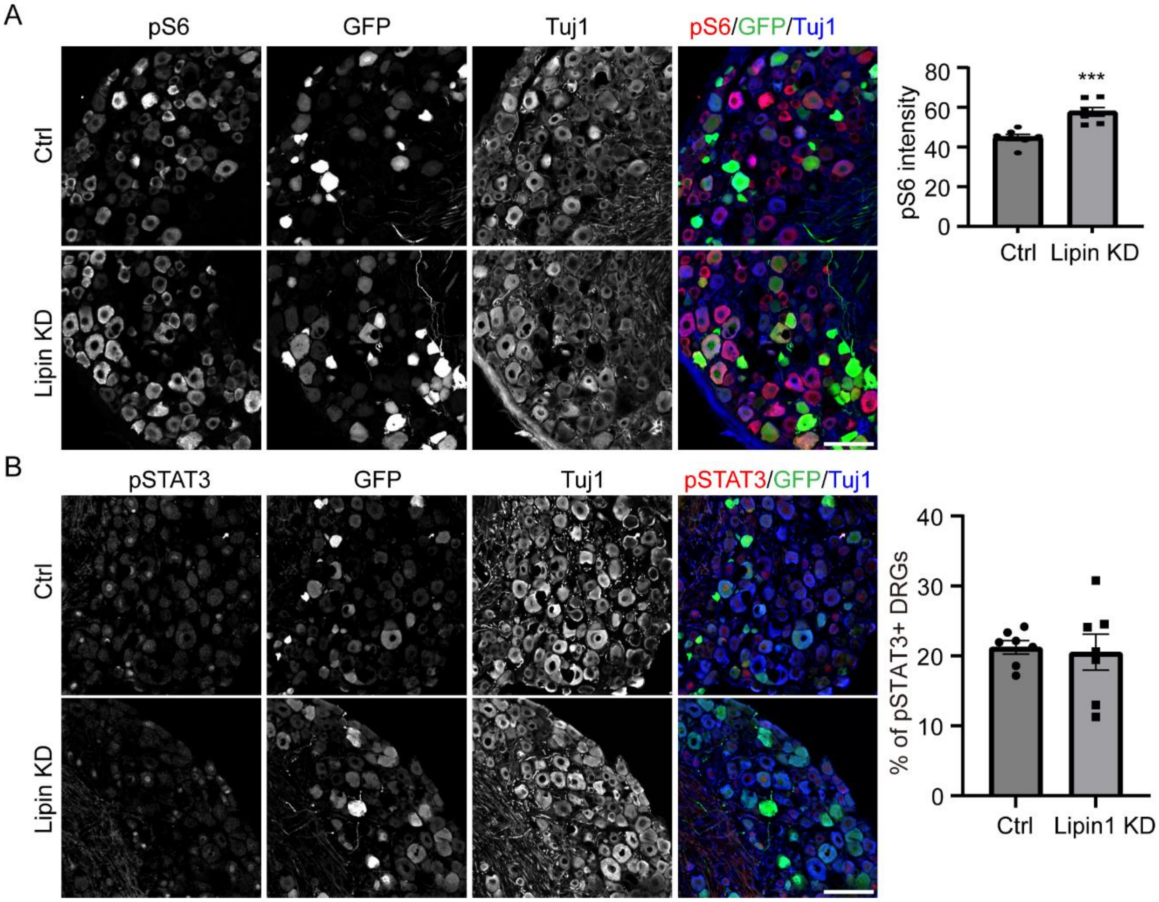

Fig. S 10. mTOR and STAT3 signaling after lipin1 KD in the DRGs. (A) Phospho-S6 level in the Ctrl and lipin1 KD DRGs. ***p≤0.001, Student's t-test. n=7 mice. Scale bar: 100 µm. (B) Phospho-STAT3 level in the Ctrl and lipin1 KD DRGs. Student's t-test. n=7 mice. Scale bar: 100 µm.

Table S1. Materials used in this study

Reagents	Resource	Identifier
Cholera Toxin B Subunit, FITC Conjugate	Sigma-Aldrich	Cat#C1655
Laminin	Gibco	Cat#23017015
Collagenase	Roche	Cat#11088858001
Sucrose	Invitrogen	Cat#15503022
DMSO	Sigma-Aldrich	Cat#D2650
Normal goat serum	Invitrogen	Cat#50062Z
Triton X-100	Sigma-Aldrich	Cat#T8787
Optimal Cutting Temperature compound	SAKURA	Cat#4583
Paraformaldehyde (PFA)	Sigma-Aldrich	Cat#30525-89-4
Neurobasal	Gibco	Cat#21103049
Fetal bovine serum	Cytiva	Cat#SH3007103
B27	Gibco	Cat#17504001
Penicillin-streptomycin	Gibco	Cat#15140122
GlutaMax	Gibco	Cat#35050061
Neurobasal-A	Gibco	Cat#10888022
HBSS	Gibco	Cat#14175079
Trypsin	Gibco	Cat#15090046
5Fdu	Sigma	Cat#F0503
DP-PA	Larodan	Cat#38-1660
PO-PA	Larodan	Cat#38-1662
P-LPA	Larodan	Cat#38-1680
O-LPA	Larodan	Cat#38-1850
CNO	Dcchemicals	Cat#DC7991

RNAscopeTM Multiplex Fluorescent Reagent Kit v2	Advanced CellDiagnostics	Cat#323100
RNAscope probe	Advanced CellDiagnostics	Cat#491161
TAK-615	MedChemExpres s	Cat#HY-117959
Ki16425	MedChemExpres s	Cat#HY-13285
AM095 free acid	MedChemExpres s	Cat#HY-16040
AzoLPA	Avanti	Cat#870626P
**Primary antibodies**		
Rabbit anti cFos	Santa Cruz	Cat# sc-52; RRID:AB_2106783
Mouse anti SMI32	Biolegend	Cat# 801701; RRID:AB_2564642
mouse anti Tuj1	Biolegend	Cat# 801202; RRID:AB_10063408
Rabbit anti pS6	Cell Signaling	Cat# 4858; RRID:AB_916156
Rabbit anti pSTAT3	Cell Signaling	Cat# 9145L; RRID:AB_2491009
Rabbit anti 5HT	Immunostar	Cat# 20080; RRID:AB_572263
Rabbit anti FITC	Invitrogen	Cat#71-1900; RRID: AB_2533978
Rat anti mCherry	Invitrogen	Cat# M11217; RRID:AB_2536611
Chicken anti GFP	Invitrogen	Cat# A10262; RRID:AB_2534023
Rabbit anti Lipin1	Santa Cruz	Cat# sc-98450; RRID:AB_2135907
Rabbit anti Lipin1	Proteintech	27026-1-AP; RRID:AB_2880727
Rabbit anti PKCγ	Proteintech	Cat# 14364-1-AP; RRID:AB_2300044

Rabbit anti GFAP	Dako	Cat# Z0334;<br>RRID:AB_10013382
Chicken anti GFAP	aves lab	Cat# GFAP<br>RRID:AB_2313547
Rabbit anti p-ERK1/2	Cell Signaling	Cat# 4370S<br><br>RRID:AB_2315112
Rabbit anti p-mTOR	Cell Signaling	Cat# 2971,<br>RRID:AB_330970
Rabbit anti mTOR	Cell Signaling	Cat# 2972,<br>RRID:AB_330978
Rabbit anti S6	Cell Signaling	Cat# 2217,<br>RRID:AB_331355
Rabbit anti β-actin	Cell Signaling	Cat# 4970,<br>RRID:AB_2223172
Rabbit anti p-AKT473	Cell Signaling	Cat# 3787S<br><br>RRID:AB_331170
**Secondary antibodies**		
Goat anti Mouse 488	Invitrogen	Cat# A11029;<br>RRID:AB_2534088
Goat anti Mouse 555	Invitrogen	Cat# A-21424;<br>RRID:AB_141780
Goat anti Mouse Cy5	Invitrogen	Cat# A10524;<br>RRID:AB_2534033
Goat anti Rabbit 488	Invitrogen	Cat# A11034;<br>RRID:AB_2576217
Goat anti Rabbit 555	Invitrogen	Cat# A-21429;<br>RRID:AB_2535850
Goat anti Rabbit Cy5	Invitrogen	A10523;<br>RRID:AB_2534032
Goat anti Chicken 488	Invitrogen	Cat# A11039;<br>RRID:AB_2534096
Goat anti Chicken 555	Invitrogen	Cat# A-21437;<br>RRID:AB_2535858

Goat anti Chicken 647	Invitrogen	Cat# A-21449; RRID:AB_2535866
Goat anti Rat 555	Invitrogen	Cat# A21436; RRID:AB_141733
Goat anti Rat 647	Invitrogen	Cat# A21247; RRID:AB_141778
**AAV Plasmids**		
pAAV.U6.shRLuc.CMV.EGFP.SV40	Penn Vector	Cat#P1867
pAAV-U6. sgRNA(SapI)_hSyn-GFP-KASH-bGH	Addgene	Cat#60958; RRID:Addgene_60958
pAAV.hSyn.eGFP.WPRE.bGH	Penn Vector	Cat# p1696; RRID:Addgene_105539
pAAV-hSyn-HA-hM3D(Gq)-IRES-mCitrine	Addgene	Cat #50463;<br><br>RRID:Addgene_50463
pAAV.hSyn.HI.eGFP-Cre.WPRE.SV40	Penn Vector	Cat#P1848<br><br>RRID:Addgene_105540
**Software and algorithms**		
Image J	https://imagej.nih.gov/ij/	RRID: SCR_002285
Prism 6	https://www.graphpad.com/	N/A
AIVIA 12	https://www.aivia-software.com	N/A

Table S2. shRNAs and sgRNAs used in this study

**shRNAs/sgRNAs**	**Sequence**
shCtrl	GACCATCAATATGACTAGA
shLipin1	CGTGTCATATCAGCAATTT
sgCtrl	GCGTCGTGACTGGGAAAACCC
sg*Pten*1	GAGATCGTTAGCAGAAACAAA
sg*Pten*2	GAAACAAAAGGAGATATCAAG

Table S3. Animal list

Experiment	Mice used	Mice excluded
Fig.1 F and Fig.1 G *mTOR* cKO	shCtrl;WT(5)+shLipin1;WT(5)+shLipin1;*mTOR* cKO(5)=15	NA
Fig. 1H and Fig. 1I *Stat3* cKO	shCtrl;WT(4)+shLipin1;WT(4)+shLipin1;*Stat3* cKO(4)=12	NA
Fig. 1J and Fig. 1K *Rptor* and *Stat3* double KO	shCtrl;WT(4)+shLipin1;WT(4)+shLipin1;*Rptor* cKO;*Stat3* cKO(4)=12	NA
Fig. S1I and S1J *Rptor* cKO	shCtrl;WT(5)+shLipin1;WT(5)+shLipin1;*Rptor* cKO(5)=15	NA
Fig. 3L and Fig. 3M Lipin1 overexpression combined with *Pten* KO	shCtrl;GFP(6)+sg*Pten*;GFP(6)+sg*Pten*;Lipin1-WT(6)=18	NA
Fig. 4B and Fig. 4D Basal sprouting	Ctrl(4)+Lipin1 KD(4)=8	NA
Fig. 4E and Fig. 4F neonatal manipulation and CST sprouting	Ctrl(6)+Lipin1 KD(6)=12	NA
Fig. 4H and Fig. 4I Adult lipin1 KD and CST sprouting	Ctrl(8)+Lipin1 KD(8)=16	NA
Fig.5C and Fig. 5D P14 spinal cord crush	Ctrl(7)+Lipin1 KD(7)=14	NA
Fig. 5F, Fig. 5G, Fig.S7, and Fig. S8 neonatal KD adult crush	Ctrl(8)+ Lipin1 KD(8)=16	NA
Fig. 5I and Fig. S8 Spinal cord clearing after regeneration	Lipin1 KD(2)	NA
Fig. 6D and Fig. 6E Regeneration of ascending sensory axons	Ctrl(7)+ Lipin1 KD(7)=14	NA
Fig. S9 adult lipin1 KD	Ctrl(7)+ Lipin1 KD(6)=13	NA

Movie S1 (separate file). Video showing continuous spinal cord slices with regenerated axons from ventral to dorsal side for animal 1.

Movie S2 (separate file). 3D reconstruction of light-sheet microscope-imaged spinal cord rotating via X axis for animal 1. The X, Y, and Z axes point to the caudal, lateral, and dorsal spinal cords, respectively.

Movie S3 (separate file). Spinal cord rotating via Y axis for animal 1.

Movie S4 (separate file). Spinal cord rotating via Z axis for animal 1.

Movie S5 (separate file). Zoom in perspective for animal 1.

Movie S6 (separate file). Video showing continuous spinal cord slices with regenerated axons from ventral to dorsal side for animal 2.

SI References

1. C. Yang, *et al.*, Rewiring Neuronal Glycerolipid Metabolism Determines the Extent of Axon Regeneration. *Neuron* (2019). https://doi.org/10.1016/j.neuron.2019.10.009.

2. F. Sun, *et al.*, Sustained axon regeneration induced by co-deletion of PTEN and SOCS3. *Nature* **480**, 372–375 (2011).

3. K. Liu, *et al.*, PTEN deletion enhances the regenerative ability of adult corticospinal neurons. *Nature Neuroscience* **13**, 1075–1081 (2010).

4. W. Chen, *et al.*, Rapamycin-Resistant mTOR Activity Is Required for Sensory Axon Regeneration Induced by a Conditioning Lesion. *eneuro* **3**, ENEURO.0358-16.2016 (2016).

5. M.-S. Yoon, Y. Sun, E. Arauz, Y. Jiang, J. Chen, Phosphatidic Acid Activates Mammalian Target of Rapamycin Complex 1 (mTORC1) Kinase by Displacing FK506 Binding Protein 38 (FKBP38) and Exerting an Allosteric Effect. *Journal of Biological Chemistry* **286**, 29568–29574 (2011).

6. J. Ellery, *et al.*, Identification of compounds acting as negative allosteric modulators of the LPA1 receptor. *European Journal of Pharmacology* **833**, 8–15 (2018).

7. H. Ohta, *et al.*, Ki16425, a Subtype-Selective Antagonist for EDG-Family Lysophosphatidic Acid Receptors. *Mol Pharmacol* **64**, 994–1005 (2003).

8. J. S. Swaney, *et al.*, Pharmacokinetic and Pharmacodynamic Characterization of an Oral Lysophosphatidic Acid Type 1 Receptor-Selective Antagonist. *J Pharmacol Exp Ther* **336**, 693–700 (2011).

9. J. Morstein, *et al.*, Optical Control of Lysophosphatidic Acid Signaling. *J. Am. Chem. Soc.* **142**, 10612–10616 (2020).

10. N. Renier, *et al.*, iDISCO: A Simple, Rapid Method to Immunolabel Large Tissue Samples for Volume Imaging. *Cell* **159**, 896–910 (2014).

11. M. Belle, *et al.*, A Simple Method for 3D Analysis of Immunolabeled Axonal Tracts in a Transparent Nervous System. *Cell Reports* **9**, 1191–1201 (2014).

12. J. Chi, A. Crane, Z. Wu, P. Cohen, Adipo-Clear: A Tissue Clearing Method for Three-Dimensional Imaging of Adipose Tissue. *JoVE* 58271 (2018). https://doi.org/10.3791/58271.

13. D. Hörl, *et al.*, BigStitcher: reconstructing high-resolution image datasets of cleared and expanded samples. *Nat Methods* **16**, 870–874 (2019).
